# Explorations of the chemical constitution and aqueous solution status of caries-arresting silver(I)-diammine fluoride and silver(I)-fluoride products using high-resolution ^19^F NMR analysis. Spectroscopic and SEM investigations of their interactions with human saliva: evidence for the *in vivo* salivary-catalysed autoconstruction of Ag/AgCl-based nanoparticles (IV-SCAN)—part I

**DOI:** 10.3389/froh.2024.1373885

**Published:** 2024-06-12

**Authors:** Kayleigh Hunwin, Georgina Page, Mark Edgar, Adolfo Botana, Rachel Armitage, Mohammed Bhogadia, Unmesh Desai, Steven Duffin, Marcus Duffin, Wyman Chan, Martin Grootveld

**Affiliations:** ^1^ Leicester School of Pharmacy, De Montfort University, The Gateway, Leicester, United Kingdom; ^2^ JEOL (U.K.) Ltd., JEOL House, 1-2 Silver Court, Watchmead, Welwyn Garden City, United Kingdom; ^3^ Shoreview Dental LLC, Keizer, OR, United States; ^4^ NoDK LLC, Wilsonville, OR, United States; ^5^ Oral Health Outreach LLC, Wilsonville, OR, United States; ^6^ SmileStudio (UK) Ltd., London, United Kingdom

**Keywords:** dental caries, human saliva, silver(I)-diammine fluoride (SDF), silver(I)-fluoride (SF), silver(I)-chloride (AgCl), chromophoric Ag/AgCl-based nanoparticles (CSNPs), IV-SCAN, mechanisms of action (MoAs)

## Abstract

**Introduction:**

Silver(I)-diammine fluoride (SDF) and silver(I)-fluoride (SF) complexes have been successfully employed for the arrest of dental caries for many years. However, to date there are very few studies available reporting on the molecular structural compositional and solution status of these agents [typically applied as highly-concentrated 38% (w/v) solutions]. Here, we explored the solution status and chemical constitution of commercially-available SDF and SF products, and secondly investigated the multicomponent interplay of these products with biomolecules present in intact human whole-mouth salivary supernatants (WMSSs) *in vitro*.

**Methods:**

High-resolution ^19^F NMR analysis was employed to explore SDF and SF product solutions, and to determine WMSS fluoride (F^−^) concentrations, whereas ammonia (NH_3_) release form SDF was tracked by ^1^H NMR spectroscopy. SEM and thin-film FTIR-ATR analyses were employed to explore the atomic and molecular compositions of sequentially-generated AgCl deposits and chromophoric Ag/AgCl nanoparticles (CSNPs); the time-dependent generation of the latter was followed spectrophotometrically.

**Results:**

^19^F NMR spectra of aqueous SF solutions contained a very broad F^−^ signal (Δv_1/2_ 70 Hz), demonstrating that much of its solvated F^−^ content was rapidly exchanging with Ag(I) on the NMR timescale, but those of SDF had a much sharper resonance, similar to that of “free” F^−^ (4 Hz). Moreover, further NMR results revealed that a popular SDF product contained high molar excesses of both F^−^ and NH_3_. Treatment of WMSSs with SDF and SF generated an off-white precipitate, which slowly developed into CSNPs at 23°C; SEM demonstrated high contents of both silver and chloride in this material (ca.1:1 atomic content ratio). FTIR-ATR analysis found that the CSNPs formed contained a range of salivary biomolecules, which appear to encapsulate the Ag/AgCl core (significant thiocyanate contents were also found). In conclusion, NMR results acquired demonstrated that SF, but not SDF, product solutions feature rapidly-exchanging F*^−^* between its “free” and Ag(I)-bound forms, and that SDF contains large excesses of both F^−^ and its NH_3_ ligands. Characterised AgCl deposits and CSNPs were sequentially produced from the interactions of these complexes with WMSS biomolecules.

**Discussion:**

In view of their well-known microbicidal and cariostatic properties, the observed autobioconstruction of CSNPs involving salivary catalysis is of much therapeutic significance.

## 1 Introduction

Dental caries represents a deterioration of teeth ascribable to adverse bacterial activities, and symptoms include pain, together with difficulties experienced with eating. Further complications involve inflammation of the tissues surrounding the tooth areas affected, tooth loss, and infection/abscess production (1). The pathogenesis of caries primarily involves bacterial-induced degradation of the hard tissues of the teeth (enamel, dentin and cementum), which arises from the demineralizing actions of organic acid bacterial catabolites, and which are generated from the consumption of food debris and/or carbohydrates located on the tooth surface. Indeed, sugars present in dietary human food sources represent the primary energy source for these bacteria, and therefore sugar-rich diets represent a major risk factor (1, 2). Hence, dental caries occurs and perpetuates if the tooth demineralization rate is greater than the remineralization process, the latter promoted by sources such as salivary calcium ions (Ca^2+^). Further risk factors include diabetes mellitus, Sjorgren's syndrome, and medications that diminish saliva production (1, 2). Moreover, dental caries is also associated with poverty, poor oral cleansing, and gum recession, phenomena which engender the exposure of teeth roots (3). This condition still serves as a significant oral healthcare challenge in the majority of industrialised countries, and is known to affect 60–90% of children, together with the great majority of adults (3). Indeed, it is highly prevalent in selected Asian and South-American countries, and pre-school children from disadvantaged communities exhibit a higher incidence of this oral disease than that of the population in general (3, 4).

Silver(I) co-ordination compounds and complexes have been therapeutically-applied in dentistry for more than 100 years, and both *in vitro* laboratory and *in vivo* clinical investigations have demonstrated their effectiveness in the prevention and arrest of dental caries in primary and permanent teeth. To date, the prime compound employed for the successful clinical arrest of dental caries is silver(I)-diammine fluoride (SDF, {[Ag(NH_3_)_2_]^+^/F^−^}); before proceeding, however, it should be noted that the commonly-used name “silver diamine fluoride” is actually a misnomer. Since silver(I) ions are near-linearly co-ordinated by two ammonia (NH_3_) N-donor ligands and not amine compounds, the correct term is “silver(I)-diammine fluoride”.

Although the adverse discolouration effects observed with the use of topically-applied SDF represent a significant problem for many patients (together with the chemical release of malodorous ammoniacal ligands from it), this form of therapy is quite easily topically applied, simple to use and offers many quite low-cost benefits for caries management. Notably, patient populations in developing third-world countries equipped only with very limited dental treatment resources and oral health services are ideally suited to this form of treatment. Similarly, institutionalised or frail elderly patient populations, again with access to only limited oral healthcare options, are also identified as target beneficiaries. Currently, SDF is used extensively in many countries for the treatment of dental caries in patients of all ages (5).

In view of the unavailability of alternative interventional approaches, treatment strategies with SDF are generally considered to be an efficient and rather facile dental application process. Multiple randomized clinical trials (with hundreds of patients involved) support its use for caries treatment, information which substantiates the success of this interventional strategy, and which therefore addresses an unmet requirement in clinical dentistry. Intriguingly, it appears that only one or two applications of SDF per annum, many of them involving the administration of only a single drop of its concentrated solution [38% (w/v)], are sufficient for the effective control of dental caries (5).

The ability of SDF to halt the dental caries process and simultaneously prevent the regeneration of new caries developments is believed to arise from the combined effects of (1) Ag(I) ion's ability to stimulate sclerotic or calcified dentin formation, e.g., (6), together with its potent bactericidal effects (7–9); and (2) the decay-arresting actions of fluoride ions (10–12). However, as we might expect, the activity of simpler silver compounds such as Ag(I)-nitrate to arrest dental caries appears to be lower than that of SDF. Hence, the enhanced activity observed for SDF may be attributable to the penetrating ability of [Ag(NH_3_)_2_]^+^ ions within carious dentin. However, SDF's mechanisms of action (MoAs) are clearly multifactorial in view of the availability of multiple microbial and sub-cellular targets for it, and the relative pathological importance of these. Indeed, since Ag(I) ion is a soft Lewis acid, the bactericidal mechanisms featured in its application may involve its powerful (inhibitory) affinity for protein cysteine thiol (-SH) functions such as that available in human collagen (such Ag(I) complexes have high formation (stability) constants). Additionally, it has an ability to coordinate to the nitrogen donor atoms of DNA base adducts and/or selected proteins/enzymes. Such processes will significantly alter hydrogen (H)-bonding and inhibit respiratory processes, DNA unwinding, cell wall synthesis and cell division (13, 14).

[Ag(NH_3_)_2_]^+^ is often synthesized at a pH value of 10.4 (cariostatic fluoride anion serves as the counter ion), and this is viewed as a favourable development in view of the critical threshold pH value at which demineralisation occurs (5.5), as reviewed in Section 4.4 below. Interestingly, some studies have suggested that the presence of Ag(I) in alkaline media enhances the formation of fluorohydroxyapatite in teeth via an Ag(I)-phosphate intermediate species (5). In point of fact, in 2017, Mei et al. (15). reported that SDF gave rise to the generation of fluorohydroxyapatite, and this observation provided evidence for its ability to directly react with calcium and phosphate ions, and in this manner give rise to the preferential precipitation of a limited solubility form of this fluoride-substituted biomineral. Hence, the development of fluorohydroxyapatite, with a diminished solubility over that of hydroxyapatite, may indeed represent a major factor involved in caries lesion arrest observed with SDF treatment.

A series of investigations have revealed that SF also serves as a very effective treatment regimen, prophylactic or otherwise, for dental caries [reviewed in (16)]. However, the mechanistic basis of these effects remain largely unexplored (16), most especially studies which consider the interactions of this agent, and SDF, with human saliva, which readily has access to it during dental caries treatment regimens.

Of particular interest, Ag(I) remains an oxidant in biosystems, since it may be reduced to Ag(0) by a range of bioavailable electron-donors [the basic redox potential of the Ag(I)/Ag(0) couple is +0.80 V (17)], although the photodecomposition of Ag(I) is readily achieved on its exposure to light. Moreover, even some unexpected reductants such as fluoride anion itself (a strong Lewis base) have the ability to donate an electron to Ag(I) to form a characteristic silver mirror in non-aqueous media such as dimethylsulphoxide or acetonitrile (18); luminescent silver nanoparticles are also generated in this reaction. This reduction process simply does not occur in aqueous solutions in view of the very high level of solvation of F^−^ by hydrogen-bonded water. A notable concern, however, is the potential instability of some concentrated solutions of Ag(I) complexes (particularly their photo-instabilities), although it is of much importance to note that in principle, these may be prepared via a one-tube “on-site” ambient temperature synthesis at the point-of-contact immediately prior to use.

Although in principle much Ag(I) will be consumed via the direct reaction of [Ag(NH_3_)_2_]^+^ with oral fluid chloride ion to form insoluble AgCl, the very large excess of this metal ion applied in clinical situations is predominantly delivered as a single drop of a 38% (w/v) SDF solution for adults. This is equivalent to a 32 µl volume (19), which is estimated to contain an application amount of 76 µmol of SDF itself (equivalent concentrations for both Ag(I) and F^−^, but two-fold higher for ammonia according to its [Ag(I)(NH_3_)_2_]^+^/F^−^ formulation) is presumably more than sufficient to allow it to exert its important bactericidal effects.

In the current paper, the authors have, for the first time, explored the interactions of SDF with a range of biomolecules available in real human saliva simultaneously, along with the release, biodisposition and fate of its Ag(I), ammonia and fluoride ion chemical constituents in this biofluid medium. Such studies are clearly of much importance in view of the ready access of this biofluid to sites of its application in the oral environment. Similar investigations were conducted with the ammonia-free anti-caries SF product. For this purpose, high-resolution ^19^F nuclear magnetic resonance (NMR), scanning electron microscopy (SEM) and attenuated total reflection Fourier-transform infra-red (FTIR-ATR) techniques were employed in our experiments. Results acquired from these studies successfully provided much valuable information regarding the biomolecular reactivities of intact SDF, along with its constituent ammonia ligands or fluoride counter ion [silver(I) and fluoride ligand/anion only for SF], with a range of salivary metabolites. Notwithstanding, a primary phase of this investigation involved an ^19^F NMR evaluation of the precise molecular nature, compositions and aqueous solution properties of the products themselves, to explore not only SDF's fluoride anion and ammonia contents, but also the solution-phase exchange of fluoride at the Ag(I) centre for both SDF and Ag(I)-fluoride products. From this study, newly-developed prospects for the therapeutic activities and MoAs of SDF, and SF complexes in general, are discussed in detail in Part II of this paper, as is relevant information focussed on the co-ordination, redox and nanoparticle chemistries of silver(I) ion, along with aspects of its potential toxicological properties.

In view of the limited information available in the scientific literature on the chemistry of silver(I) and SF complexes, particularly their relevant bioinorganic chemistry, we have also included two Sections in our Part II paper,^1^ the first featuring an overview of silver coordination chemistry, the second involving an account of the aqueous solution status and equilibria of SF complexes.

## 2 Materials and methods

### 2.1 ^19^F NMR analysis of commercially-available SDF and silver(I)-fluoride clinical formulations

The SDF and SF product solutions evaluated in this study mainly featured those intended for clinical use: Riva Star, Advantage Arrest and Fagamin products purchased from or supplied by SDI Ltd., Elevate Oral Care LLC and Tedequim SRL respectively. Unless otherwise indicated, all experiments conducted with SDF, SF and silver(I)-nitrate were conducted with protection against light, including the use of darkened glass or plasticware.

At the macro-analysis level, commercial formulation samples of both SDF and ammonia-free 1:1 SF products for clinical application were prepared in doubly-distilled water following their dilution to concentrations of 59.8 mmol/L; as specified by the manufacturer, the original SF component concentrations of these agents were both 38% (w/v), yielding a molar concentrations of 2.99 mol/L. Following the addition of 10% (v/v) ^2^H_2_O as a field frequency lock, these samples underwent ^19^F NMR analysis to explore the solution status of the molecular nature of F^−^ anion in these samples, i.e., the solution exchange properties of this anion at the Ag(I) centre, and/or its ability to form ion-pair complexes with this metal ion. For comparative purposes, stock solutions of sodium fluoride in HPLC-grade water containing 10% (v/v) ^2^H_2_O were prepared at concentrations ranging from 10 to 600 mmol/L. For all these samples, pH values were not adjusted with phosphate or other buffering agents in view of the likely complexation/chelation and/or precipitation of Ag(I) by such agents. Likewise, the sodium trifluoroacetate (TFA) quantitative internal standard was not added to these samples in case of its possible complexation of Ag(I) ion. A total of *n* = 3 replicate solutions of each product was analysed in this manner.

Further experiments focused on determinations of the fluoride levels in these commercial products only involved the ^19^F NMR analysis of diluted samples of them (1/5, 1/10, 1/30 and 1/200 dilutions) in the same 10% (v/v) ^2^H_2_O medium, which also contained appropriate concentrations of TFA (Fisher Scientific, Loughborough, Leicestershire, UK) added to them as a quantitative internal standard (final added levels of 1.08–10.80 mmol/L). Coefficients of variation (CVs) of replicate F^−^ determinations made on the same samples were found to range from 3.8% to 5.8%.

Additional ^19^F NMR experiments were conducted on aqueous solutions of commercial Ag(I)-fluoride products (Sigma-Aldrich, St. Louis, MO, USA) which were for synthetic organic chemistry use and not at all intended for therapeutic applications in dentistry. For this purpose, a 500 MHz JEOL spectrometer was employed operating at a frequency of 470.4 MHz for ^19^F. This spectrometer was also used to acquire ^19^F NMR spectral profiles on aqueous solutions of several different commercially-available SDF products for clinical dental application. Spectra acquired (on *n* = 3 replicates) were processed without backward linear prediction, and with a line broadening of 10 Hz in order to clearly observe very broad signals which were absent from blank samples.

The commercial clinical sample of 38% (w/v) SF was quite strongly acidic. However, very large dilutions of it in HPLC-grade water yielded pH values of ca*.* 5.0–5.5, which was found to be quite sufficient to protect against its decomposition during the course of standard calibration experiments conducted here.

### 2.2 Collection and preparation of human saliva samples for NMR, SEM and FTIR-ATR analyses

Experiments performed on the interaction of SDF with human saliva samples predominantly involved only one of the different clinical products available. Methods employed for experiments focused on analyses of fluoride counter-ion and ammonia ligand present in this product, by ^19^F and ^1^H NMR spectroscopies, respectively, are outlined in Sections 2.3, 2.4 and 2.7 below respectively. Unstimulated human saliva specimens were collected from a total of *n* = 12 healthy adult volunteers (8 females and 4 males, mean ± SD age 28 ± 2.4 years). Notwithstanding, some of these participants donated up to three different samples for this study on different sampling days via informed consent. All samples were numerically- and randomly-coded prior to their preparation for ^1^H NMR analysis, and hence samples were anonymised. In order to avoid any interferences arising from the introduction of exogenous agents into the oral environment, all volunteers were requested to refrain completely from oral activities (i.e., eating, drinking, cigarette smoking, tooth-brushing, oral rinsing, etc.) for a period of at least 8 h prior to sample collection, as previously described (20). Participants were directed to collect all saliva available (ca. 2–8 ml), i.e., “whole” saliva expectorated from the mouth, into a sterile plastic universal tube. These whole mouth saliva specimens were then rapidly transported to the laboratory on ice, and then centrifuged immediately in order to remove cells and debris. The resulting supernatants, abbreviated WMSSs, were then stored at −70°C for a maximum duration of 30 h. prior to analysis using the different techniques outlined below.

All studies involving human WMSS samples were conducted according to the guidelines of the Declaration of Helsinki. The investigations outlined were approved by the Research Ethics Committee of The Faculty of Health and Life Sciences, De Montfort University (DMU), Leicester (reference no. 457249). WMSS samples had a mean ± SD pH value of 6.91 ± 0.17, and these were determined with a hand-held Accument AET 15 pH meter (Fisher Scientific Ltd., Loughborough, UK).

### 2.3 Treatment of WMSS samples with SDF, and preparations of solution-phase supernatant and nanoparticulate deposit samples for ^1^H and ^19^F NMR, FTIR-ATR, spectrophotometric and SEM analyses

Commercially-available concentrated SDF and SF solutions for clinical use from differing manufacturers were purchased from dental supplier retail outlets based both in the USA and the UK. For the ^19^F NMR studies conducted, aliquots (10.0 µl) of a 1/10 dilution of the “neat” clinical application solution of an SDF product [containing 35%–40% (w/v) of the SF component, which is equivalent to 2.76–3.15 mol/L according to the manufacturer's specifications] in HPLC-grade water were added to 0.70 ml volumes of human WMSSs, specifically those collected from *n* = 5 participants, with *n* = 3 replicate samples collected from each one. This treatment primarily generated an off-white silver(I)-chloride-type precipitate. Following thorough vortex mixing and centrifugation at 10.000 rpm and 4°C, sufficient volumes of the resulting clear supernatant were then removed for ^19^F NMR analysis. To these samples was added TFA as an internal standard (*δ_F_* = −75.3 ppm) at a final added concentration of 1.08 mmol/L for ^19^F NMR analysis, or the 3-(trimethylsilyl)propionate-2,2,3,3-d4 (D 98 atom%) internal standard (*δ* = 0.00 ppm) added at a final level of 238 µmol/L for the ^1^H NMR analysis of NH_3_ (as NH_4_^+^, Section 2.7), subsequent to sample preparation.

Corresponding small microlitre additions of a 1/10 dilution of the most alkaline commercial SDF product investigated in HPLC-grade water (pH 10.5) to WMSS samples exerted little or no effect on their pH values (monitored on samples still containing the preliminary off-white AgCl precipitate); indeed, these only increased by up to 0.4 pH unit, and this confirmed the quite strong buffering capacity of this biofluid (however, addition of 5 µl of the undiluted, neat SDF product to 2.00 ml volumes of WMSS samples did result in a significant pH rise). Likewise, treatment of WMSS specimens with corresponding small volumes of a 1/10 dilution of the acidic commercially-available SF product examined in HPLC-grade water was found not to significantly reduce their final pH values.

For the semi-quantitative ATM-FTIR investigations, WMSS samples were again primarily thoroughly centrifuged at 10,000 rpm and 4°C for a period of 5 min. The clear WMSSs were then removed, and 0.10 and 0.80 ml aliquots of these were transferred into separate Eppendorf tubes, the first serving as an untreated control. The second 0.80 ml sample aliquot was then treated with 10 µl of a 1/10 diluted aqueous solution of the “neat” commercial SDF formulation, and then both samples were again vortex mixed and centrifuged for 5 min. as described above. The clear supernatants were subsequently transferred to further unused Eppendorf tubes and stored in the dark at 4°C whilst awaiting analysis. Following removal of the clear supernatant, the white/pale grey deposit remaining, which arose from the addition of SDF to human WMSS samples, was thoroughly washed three-fold with ice-cold HPLC-grade water, and samples were then left to air dry in a dark laboratory environment at ambient temperature for both SEM and ATM-FTIR analyses (usually for >16 h. overnight).

### 2.4 ^19^F NMR analysis of WMSS samples acquired prior and subsequent to the addition of diluted SDF solution products for clinical use

^19^F NMR analysis of WMSS samples collected from *n* = 5 participants (with four of them donating three separate replicate samples, and one of them providing two) was performed before and after their *in vitro* treatment with SDF as described above. Participants were coded A–E. Analysis solutions contained 527 µl WMSS samples, 60 µl of ^2^H_2_O, and 13 µl of a 50.00 mmol/L TFA internal standard (*δ_F_* = −75.3 ppm). ^19^F NMR spectra were acquired on a JEOL JNM-ECZR 600 MHz NMR spectrometer operating at a frequency of 564.72 MHz for ^19^F, throughout a 400 ppm chemical shift range width, with an FID acquisition time of 0.92 s. A 90^°^ pulse (8.3 µs) was employed, with a 3 s relaxation delay between pulses. In total, 2,048 scans were acquired with 256K data points, which were then Fourier-transformed with zero-filling to 512K data points and a single exponential function of 5.0 Hz. The interfering baseline roll signal from the fast-relaxing fluoropolymer present in the NMR probe-head was removed using backward linear prediction (order = 16, sample data 512 points, reconstructed data 32 points). A polynomial function was applied for the purpose of baseline correction. Chemical shifts were referenced to external fluorotrichloromethane (CFCl_3_, the JEOL UK Ltd. default spectrometer reference setting). Standard calibration curves were generated using a series of sodium fluoride standards with ranges of 0–200 µmol/L for untreated (control) WMSS samples, and also 0–10 mmol/L for SDF-treated samples analysed subsequent to removal of the Ag/AgCl nanoparticulate deposits formed.

For experiments conducted with SDF, 0.60–0.70 ml volumes of WMSS samples were treated with small microlitre aliquots of a 1/10 diluted commercial product solution of SDF for clinical use. Following removal of the Ag/AgCl-containing precipitate via centrifugation, 0.50–0.60 ml aliquots of the clear supernatant sample deriving therefrom was removed for ^19^F NMR analysis. From the determined F^−^ content of the SDF product employed, estimated levels of this agent in these samples were 4.58 mmol/L, and known added TFA concentrations were 1.08 mmol/L. CVs for replicate F^−^ determinations made on WMSSs with this technique were 4.1% ± 1.5% (mean ± SD).

In a further experiment designed to investigate the precise chemical shift and Δv_1/2_ values, WMSS samples were collected from a further seven participants (4 female/3 male, mean ± SD age 26 ± 2.7 years), and prepared for SDF treatment and ^19^F NMR analysis as described above. Following the addition of the 1/10-diluted clinical SDF solution (10 µl) to a 0.70 ml aliquot of WMSS samples, vortex mixing and thorough centrifugation (Section 2.3), the clear supernatants arising therefrom underwent ^19^F NMR analysis as described above. Triplicates of each of the seven SDF-treated WMSS samples were individually prepared for ^19^F NMR analysis, and analysed as such.

Provisional ^31^P NMR investigations were performed on WMSSs, along with acidic (pH 2.00) extracts of the nanoparticulate deposit formed on addition of SDF to these samples, using the above spectrometer operating at a frequency of 242.88 MHz. Similarly, provisional ^15^N NMR studies performed on chloroform extracts of the SDF and SF products, the latter for both clinical and non-clinical uses, were also acquired on this spectrometer (operating frequency 60.80 MHz).

### 2.5 SEM analysis of Ag/AgCl nanoparticulate samples generated from the treatment of human WMSS samples with SDF

SEM analysis of the Ag/AgCl-based nanoparticulate precipitate obtained from a total of *n* = 3 WMSS specimens, each provided by a different donor (Section 2.3), was performed using a Carl Zeiss EVO LS 15 facility, equipped with an Oxford Xmax 80 mm^2^ EDS detector. An accelerating voltage of 5 kV, and a 20 µm aperture were employed for this purpose. The images were taken using secondary electron (SE) and back-scattered electron (BSD) detectors, and the energy dispersive spectroscopic (EDS) maps acquired were contrasted using 20 frames.

### 2.6 FTIR-ATR analysis of WMSS samples prepared before and after SDF treatment, and that of Ag/AgCl nanoparticulates generated therefrom

FTIR-ATR analysis of WMSS samples (three daily replicates collected from *n* = 5 different participants) was conducted using an ALPHA II-Platinum FT-IR spectrometer equipped with a universal platinum diamond-ATR QuickSnap sampling module (this compact spectrometer permits rapid, simple and reliable FTIR analysis). Firstly, the spectrometer sample holder window was cleansed with an alcohol spray, and when removed a background spectrum was recorded. Subsequently, 7.0 µl aliquots of WMSS samples were placed onto the detection window and then left to dry completely as a thin film on the spectrometer window for a minimum period of 20 min. Spectra were then acquired using a resolution of 2 cm^−1^, a measurement time of 20 s, and a scan range of 400–4,000 cm^−1^ with atmospheric compensation. The interferogram size was 30,402 points. Each sample was analysed in triplicate. This analysis was also performed on residual WMSS samples isolated via centrifugation from the Ag/AgCl nanoparticulate deposit formed on SDF addition to the intact samples (3 separate daily replicates collected from *n* = 5 different sample donors).

There was a high level of reproducibility between triplicate analyses of the same WMSS samples using this technique. To determine this, metabolomics spectral homogeneity indices were computed. For this purpose, the constant sum-normalised intensities of 100 different FTIR transmission values spread throughout the 500–2,400 and 2,600–3,700 cm^−1^ wavenumber ranges were measured, and then compared “between-replicates” using a one-way completely randomized design ANOVA model for each wavenumber selected. From this analysis, no significant “between-replicate” differences were found for all wavenumber comparisons made; the Bonferroni correction for multiple sample comparisons was adopted, although raw *p* values of >0.20 were obtained for all tests conducted.

FTIR-ATR analysis of replicates of the thoroughly washed and dried solid-phase Ag/AgCl nanoparticulate samples generated (*n* = 5) were similarly performed via the placement of these deposits onto the spectrometer window, and this analysis was also conducted in triplicate for each sample.

### 2.7 ^1^H NMR determination of the release of ammonia ligands from SDF following its addition to WMSS samples

Ammonia (NH_3_) was determined in *n* = 3 different human WMSS samples both before and after the addition of SDF in order to estimate the release of this ligand from the near-linear two-coordinate [Ag(NH_3_)_2_]^+^ complex. For this purpose, 10.0 µl of the 1/10 diluted SDF solution [containing 3.8% (w/w) of this agent according to the manufacturer's specified original sample “neat” content, equivalent to 0.236 mol/L] was added to 0.70 ml volumes of WMSS samples (*n* = 3). Samples were then thoroughly vortex-mixed and centrifuged (10 min at 4°C) and the clear residual WMSS removed. Aliquots (0.10 ml) of these samples were then treated with 70 µl of a 2.50 mmol/L stock solution of tetra-deuterated trimethylsilylpropionate (TSP, quantitative internal standard and ^1^H NMR chemical shift reference, *δ* = 0.00 ppm) in ^2^H_2_O (the latter serving as a field frequency lock), and 15 µl of 1.00 mol/L HCl, and these analyte solutions were each made up to a final volume of 0.70 ml with HPLC-grade water. Samples were then subjected to ^1^H NMR analysis using our recently developed method which determines ammonia (NH_3_) as ammonium ion (NH_4_^+^) in WMSS samples at a pH value of 2.00 (21). A corresponding analysis of the NH_3_ content of the SDF commercial product employed for these studies was also conducted by this ^1^H NMR method with HPLC-grade water used in place of the collected and prepared WMSS samples.

### 2.8 Time-dependent development of Ag/AgCl nanoparticulate species in silver(I) ion-treated WMSS samples at ambient temperature with and without exposure to a light source

For these experiments, stock solutions of known concentrations of silver(I) nitrate, SF and SDF (ca. 100 mmol/L) were prepared and stored in the dark in a refrigerator at 4°C, and when ready for use, microlitre aliquots of these were added to WMSS samples so that their final concentrations were 1.00 and 10.00 mmol/L. For this part of the study, three participants each donated *n* = 3 separate saliva samples, and all of these were utilized in the experiments involved.

Added reducing agents such as borohydride were not required for nanoparticle generation in this study since WMSS samples were found to contain sufficient levels of electron-donors to allow their formation, and the nanoparticles were allowed to develop over increasing periods of time at ambient temperature (23°C). Photographic representations and electronic absorption spectra of these were obtained at the 24.0 h post-equilibration time-point for both light-exposed and -unexposed samples. Electronic absorption spectra were acquired on a Thermoscientific Evolution 201 UV-Vis. spectrophotometer operating in the 200–600 nm range with a scan speed of 120 nm/min. Samples were analysed in a quartz cuvette with a pathlength of 1 cm (the absorbance threshold was set at 0.01). The spectrophotometer was calibrated using a blank solution of HPLC-grade water prior to each spectral acquisition. All samples collected before, during and after the equilibration process were analysed in triplicate. Electronic absorption spectra of 1/10 and 1/50-diluted samples of 1 mg/ml (7.87 mmol/L) SF standard solutions were similarly obtained.

### 2.9 Statistical analysis

Descriptive statistics were computed, and both one- and two-way (ANOVA) analysis (completely randomized and randomized blocks designs respectively) were conducted, using *XLSTAT2020* software options (Addinsoft, Paris, France).

## 3 Results

### 3.1 Solution structure, status and fluoride contents of commercial SDF and SF products: ^19^F NMR investigations

**Figure 1:**
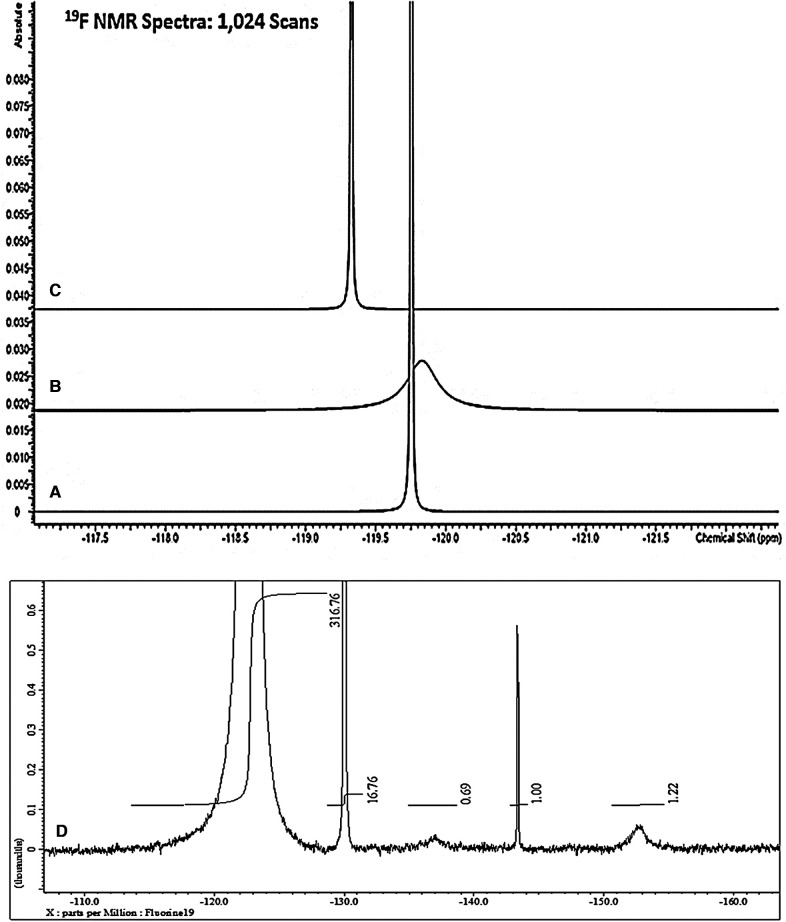
564.5 MHz ^19^F NMR spectra acquired on aqueous solutions of (**A**) sodium fluoride (60.0 mmol/L); (**B**) 1:1 silver(**I**)-fluoride (Ag(**I**)-F, (59.8 mmol/L); and (**C**) silver(**I**)-diammine fluoride (SDF, 59.8 mmol/L). Solutions were prepared in HPLC-grade water containing 10% (v/v) ^2^H_2_O. (**D**) Corresponding 470.4 MHz spectrum of a commercial sample of 1:1 Ag(**I**)-fluroide not intended for clinical use, and this was acquired in aqueous solution at a concentration of 1.00 mol/L. The outlines display electronic integration profiles and their intensities, with the major broad Ag(**I**)-fluoride signal at −122.6 ppm having a value of 317.

Figure 1 shows the ^19^F NMR profiles acquired on solutions of (a) sodium(I) fluoride (60.0 mmol/L), (b) 1:1 SF (59.8 mmol/L as specified SF level) and (c) SDF [59.8 mmol/L as specified original Ag(I)-fluoride level] in water containing 10% (v/v) ^2^H_2_O. As noted above, buffer was not added to these samples in view of the ability of such agents, e.g., inorganic phosphates, to compete for, complex and precipitate Ag(I) ions. However, since the ^19^F chemical shift value (*δ_F_*), and its line-width at half-height (Δv_1/2_) are known to be largely invariant to pH at values >5.2 (22), from the spectra displayed, the pH values of all the diluted clinical SDF and SF samples evaluated were clearly greater than this threshold value. This is expected for SDF samples which all have mildly or even higher alkaline pH values, but we have found that selected SF solutions utilized for clinical application are indeed quite highly acidic. Indeed, the pH value of replicate 1/100 dilutions of one SF product tested was as low as 4.41 ± 0.01 (mean ± SD). In view of this observation, the pH value of the “neat” product solution is expected to be considerably lower. Hence, ironically, this low pH value of the product evaluated indicates that it may adversely contribute towards the tooth demineralisation process, and this is discussed in Section 4.

These results clearly show that SF has a much broader ^19^F NMR signal (Δv_½_ 70.59 ± 1.12 Hz) than those of both free fluoride (Δv_1/2_ 3.42 ± 0.16 Hz) and SDF (Δv_1/2_ 3.92 ± 0.42 Hz). Hence, these data acquired provided strong evidence for a rapid exchange of F^−^ at the Ag(I) centre on the ^19^F NMR timescale for SF in aqueous solution, but not so for SDF, since it is already complexed by two monodentate NH_3_ ligands.

Figure 1D shows an expanded ^19^F NMR spectrum of a commercially-available Ag(I)-fluoride material for organic synthesis (non-clinical) applications as purchased from a chemical retailer. In addition to the broad F^−^ signal attributable to its rapidly-exchanging form at the Ag(I) metal ion centre (*δ_F_* = −122.6 ppm, Δv_½_ = 67 Hz), resonances located at −130.0, −137.0, −143.4 and −153.0 ppm are also clearly visible, those at −137.0 and −153.0 being very broad (Δv_½_ = ca. 500 Hz for both). These signals are therefore presumably ascribable to two further forms of Ag(I)-exchangeable F^−^ anion, the latter being assignable to F_2_H^−^ (18), although as reported, this resonance is already quite broad without the involvement of Ag(I) exchange, and this broadness possibly arises from an H-bonding F^−^ exchange with HF instead. The integrated intensity of the −153.0 ppm signal was only 0.4% of that of the broad Ag(I)-fluoride one at −122.6 ppm. However, the signal at −137 ppm remains unassigned in this report, and future studies will be performed by the authors in order to determine its precise identity.

However, the much sharper resonances centred at *δ_F_* = −130 and −143.4 ppm (Δv_½_ = 1.1 and 4.0 Hz respectively) are ascribable to hexafluorosilicate, and one or more of its hydrolytic products, and boron tetrafluoride (BF_4_^−^) anion and/or its derivatives, respectively. These species arise from the ability of quite concentrated Ag(I)-fluoride derivatives in aqueous solution, specifically the Ag(I)-F_2_H complex, to attack and “etch” borosilicate glassware (23) utilized for the commercial production of the NMR tubes utilized in the current study (these results will be reported in detail elsewhere). This is the reason why it is not recommended that Ag(I)-fluorides and their derivatives are stored in, or come into contact with, such glassware, especially at acidic pH values, and most especially for samples and materials to be employed for clinical application.

As expected, Δv_½_ values of three further different types of commercial SDF products for direct dental applications were 3.3, 5.7 and 7.0 Hz, respectively, values which again were much lower than that observed for Ag(I)-fluoride (67 Hz), which was very similar to that observed in Figure 1B (70.59 Hz). The Δv_½_ value for aqueous sodium fluoride was only 3.4 Hz for these studies conducted at an operating frequency of 470.4 MHz. Although the *δ_F_* values of the F^−^ signals for the three different SDF products tested were very similar (−116.7, −118.4 and −118.5 ppm), the spin-lattice relaxation time (*T*_1_) value of one of these differed quite significantly from that of the remaining two products, i.e., 12.0 vs. 5.0 and 4.6 s, and these data indicate that F^−^ present in the first product appears to have a relaxation behaviour which is distinct from those of the other two assessed. Corresponding Δv_1/2_ values for the 1.08 mmol/L trifluoroacetate internal standard employed in Section 3.2.3 below (*δ_F_* = −75.3 ppm) was only 2.3 Hz.

Hence, these aqueous solution-based ^19^F NMR investigations provided much evidence for fast exchange of the F^−^ ligand on the NMR timescale for Ag(I)-fluoride solutions; indeed, a very broad ^19^F NMR resonance was observed for this complex in its clinically-applied solution, its line-width at half-height being substantially larger than that of corresponding solutions of SDF. Moreover, the line-width of “free” [i.e., non-Ag(I)-complexed] fluoride was also much lower than that found for Ag(I)-F, as expected (Figure 1A). Additionally, ^19^F NMR evidence for the complexation and further exchange of excess F^−^ by Ag(I) ion was also obtained [added F^−^ concentration-dependent modifications in its ^19^F chemical shift value were also observed when it was ^19^F NMR-spectrally titrated against the 1:1 SF agent (data not shown)]. In further provisional experiments, the “neat” SF product containing 35%–40% (w/v) (2.76–3.15 mol/L) of this agent, (according to the manufacturer), was diluted to a final concentration of 59.8 mmol/L, and a temporal (time-dependent) evolution of ^19^F NMR profiles was acquired on rotamixed samples at a temperature of 23°C immediately following dissolution, and then at 30 min intervals thereafter up to a final time-point of 360 min. Interestingly, it was possible to view both time-dependent modifications in the ^19^F NMR chemical shift parameter of this complex (from *δ_F_* = 125.4 to 121.87 ppm), together with significant decreases in its Δv_½_ value (from >500 to 95 Hz). These decreases both appeared to follow a first-order decay process (*δ_F_* and Δv_½_ were 124.2 ppm and 370 Hz respectively at the 30 min time-point); spectra of samples analysed at the 24 h post-dilution time-point had final Δv_½_ values of ca. 70 Hz, values similar to that observed in Figure 1B (data not shown). The temporal decreases in line-width indicated increases in the *T*_2_ value of this complex arising from a decrease in the extent and rate of the fast exchange of F^−^ at the Ag(I) metal ion centre. However, in addition to this phenomenon, that observed for its *δ_F_* value is also likely to reflect time-dependent, dilution-mediated increases in the pH value of the product medium (24), a process which may also involve F_2_H^−^ as an Ag(I) ion complexant, most especially in the undiluted high concentration product itself, and also at the earlier time-points. These data will be reported in detail in an upcoming future publication.

**Figure 2:**
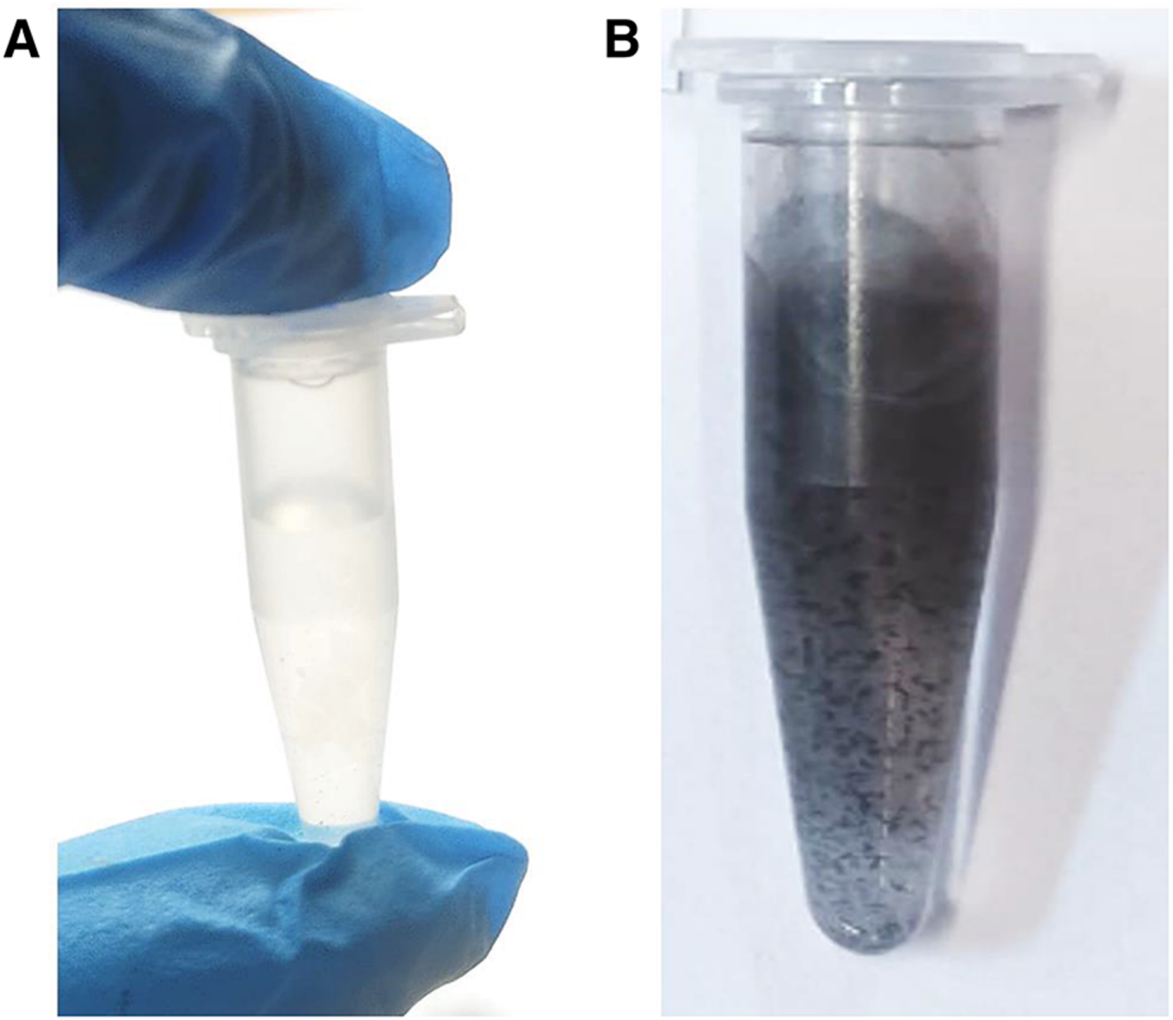
Photographic representation of aqueous solutions containing a non-clinical SF sample (0.10 mol/L) equilibrated for a duration of 10 days at 23°C and pH values of (**A**) 5.40 and (**B**) 7.00 following storage in the dark for a duration of 10 days. Discolouration and precipitate deposition of the pH 7.00 solution commenced immediately following its preparation.

Since our experiments have also shown that adjustment of the pH of 1:1 SF solutions for non-clinical use to values of <5.5 exerts a marked stabilising effect on this agent, the high acidity of the commercial SF product evaluated is undoubtedly responsible for its enhanced stability, specifically its resistance to hydrolysis, a process which in turn engenders the deposition of insoluble hydrolysis and redox reaction products from solution. Conversely, in aqueous solution at or close to neutrality, SF is actually very unstable, and the deposition of dark brown/black-coloured silver(I)-oxide and metallic silver occurs (Figure 2), even if such solutions are stored unexposed to light.

### 3.2 Interactions of SDF, Ag(I)-fluoride and Ag(I)-nitrate with human saliva

#### 3.2.1 Nanoparticulate formation on treatment of WMSS samples with Ag(I) complexes

**Figure 3:**
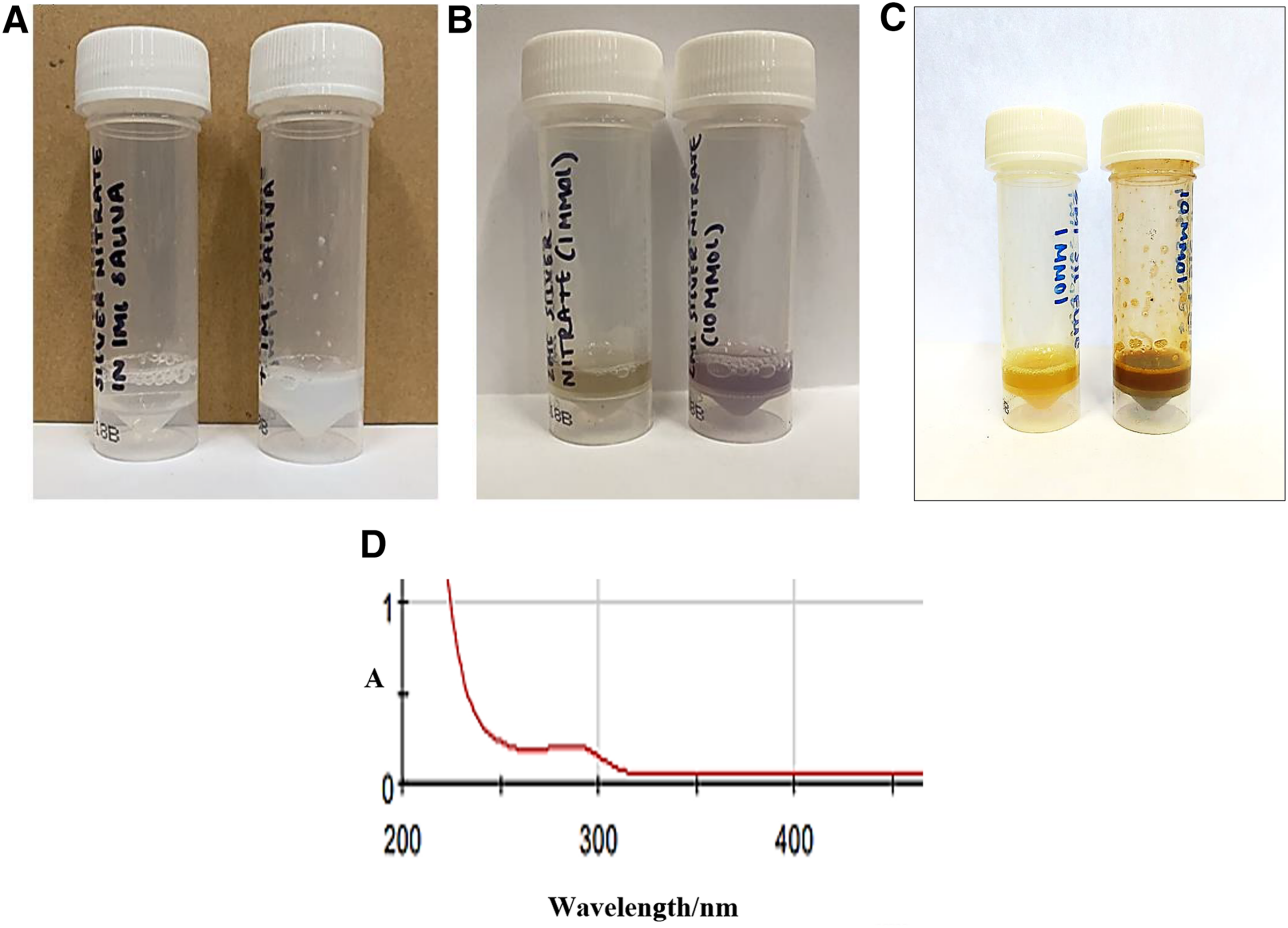
Photographic representations of Ag/AgCl nanoparticles formed after addition of Ag(**I**)-NO_3_ and Ag(**I**)-F to human WMSS samples (silver(**I**) compounds were purchased from a chemical company, and were intended for reagent and not clinical application). Photographs were obtained following (**A**) dark and (**B**) light storage for added Ag(**I**) concentrations of 1.00 (left) and 10.00 mmol/L (right) silver(**I**)-nitrate respectively form a duration of 24.0 h (yellow and red/purple colourations were visible for added levels of 1.00 and 10.00 mmol/L, respectively); (**C**), as (**B**), but with Ag(**I**)-F added in place of Ag(**I**)-NO_3_; (**D**) electronic absorption spectrum of a 1/5-diluted sample of light-exposed WMSS treated with 1.00 mmol/L Ag(**I**)-NO_3_, the colouration being allowed to develop at ambient temperature for a period of 24.0 h.

The addition of small microlitre aliquots of dilute aqueous Ag(I)-nitrate or Ag(I)-fluoride solutions to WMSS samples of volume 0.60–1.00 ml, so that the final added Ag(I) level was either 1.00 or 10.00 mmol/L, both gave rise to the immediate generation of a white/grey AgCl precipitate, which following storage in the light for a duration of 24.0 h at ambient temperature gave rise to a concentration-dependent generation of CSNPs (Figure 3). Indeed, the 1.00 and 10.00 mmol/L Ag(I)-treated samples generated yellow- and red/purple-coloured solutions, respectively, when stored with exposure to light. However, those equilibrated in the dark for the same period at this temperature remained a white/grey-white appearance during this storage period, but subsequent to this slowly developed these characteristic and quite intense yellow and then red/purple nanoparticle colourations. Following this, electronic absorption spectra were acquired on the CSNPs produced, and these revealed the development of an intense absorption band centred at ca. 280–290 nm. However, since this band was very broad, it extended quite some way into the visible region of the electromagnetic spectrum (Figure 3D). These results are consistent with the reported optical absorption spectra found for biosynthesized silver nanoparticles in previous investigations conducted (25, 26). Similar results were observed following the addition of SDF to WMSS samples. WMSS electron donors available for the reduction of Ag(I) to Ag(0) during CSNP development include thiols such as L-cysteine, thiocyanate anion (SCN^−^), and the phenolic amino acid L-tyrosine, and their availabilities and potential activities in this context are outlined in Section 4.

#### 3.2.2 SEM analysis of the elemental compositions of Ag/AgCl nanoparticulate deposits formed from the reaction of SDF with WMSS chloride and further biomolecules

Determination of the elemental compositions of the Ag/AgCl nanoparticulate deposits was subsequently performed by SEM analysis. These results confirmed the successful formation of Ag/AgCl nanoparticles with predominant elements of silver (49%–72%), chlorine (13%–20%), carbon (5%–14%) and oxygen (2%–13%) by weight. The intense Ag and Cl peaks confirmed relatively high Ag and Cl contents, and our findings have been corroborated by reports already available for the biosynthesis of similar materials from “green” plant sources (27, 28).

##### 3.2.2.1 Ag/AgCl nanoparticle size distribution, morphology and elemental abundance

**Figure 4:**
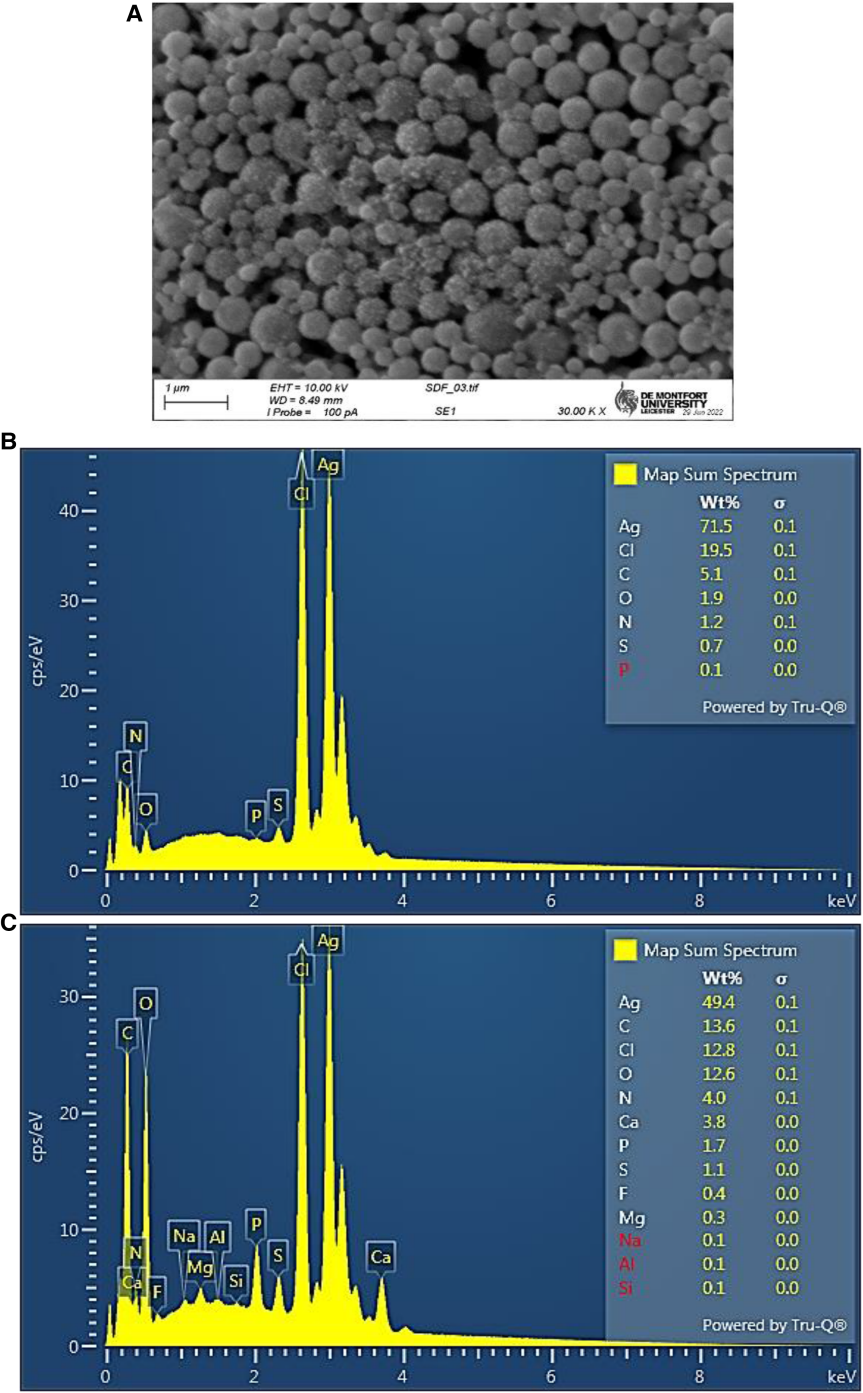
(**A**) Scanning electron microscopy (SEM) micrograph of Ag/AgCl nanoparticulates. (**B**,**C**), SEM estimation of the elemental abundance of two separate samples, showing high contents of both silver and chlorine (elemental content weight percentages are shown).

SEM analysis confirmed the presence of Ag/AgCl nanoparticles, and these mainly had a spherical shape (Figure 4A). From the image analysis, these nanoparticles predominantly had diameter sizes ranging from 50 to 300 nm, with most values being <200 nm. However, larger particles with diameters of >300 nm were also observable.

From the elemental weight percentages determined (Figures 4B,C), computed mean ± SD values for the atomic composition of these Ag/AgCl nanoparticulate deposits were silver 24.9% ± 15.3%, chlorine 20.0% ± 12.6%, carbon 28.9% ± 8.4%, oxygen 15.4% ± 12.7%, nitrogen 6.7% ± 3.0%, calcium 1.5% ± 2.1%, sulphur 1.4% ± 0.3%, phosphorous 0.6% ± 0.6%, fluorine 0.3% ± 0.55%, and magnesium 0.2% ± 0.3%. These data confirm that the atomic percentages of both silver and chlorine were almost equivalent to each other, i.e., a 1:1 ratio, with a small excess (ca. 25%) of silver which may represent the metallic Ag(0) elemental fraction present (although quite large differences were observed between both the Ag and Cl contents between the samples evaluated, the Ag:Cl atomic content ratios of these were very similar at 1.24 and 1.27). The presence of low but nevertheless significant levels of sulphur (>1.0 atomic %) presumably reflects the incorporation of SCN^−^ anion in these colloids, as confirmed by FTIR-ATR analysis described below in Section 3.2.5.

#### 3.2.3 ^19^F NMR analysis of WMSS samples before and after *in vitro* treatment with SDF

**Figure 5:**
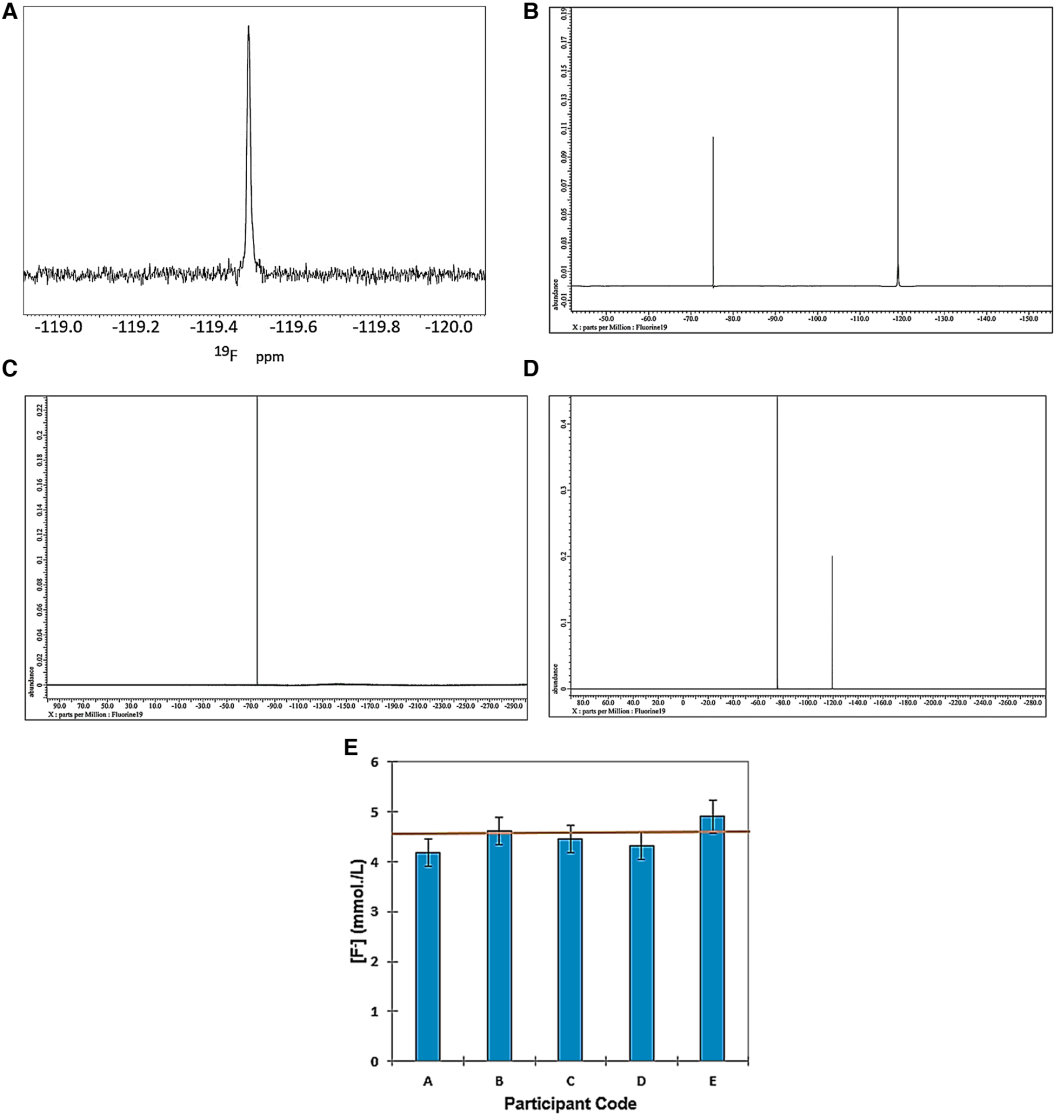
^19^F NMR spectra of aqueous standards and WMSS samples with and without treatment with SDF. (**A**) Fluoride naturally present in a typical WMSS sample with an estimated concentration of 16.4 µmol/L (expanded −120 to −119 ppm *δ_F_* region only); (**B**) an HPLC-grade water solution following the addition of a microlitre aliquot of a 1/10 diluted sample of SDF (final estimated F^−^ concentration 4.58 mmol/L); (**C**) WMSS without the addition of a diluted SDF solution; (**D**) as (**C**), but following the addition of a microlitre aliquot of a 1/30-diluted commercially-available clinical SDF product solution, removal of the discoloured Ag/AgCl precipitate via centrifugation, and spectral acquisition following treatment with the TFA internal standard; (**E**) plot of mean ± 95% CIs for determinations of F^-^ anion in WMSS samples following the addition of 10 µl of a 1/10-diluted SDF product solution (*n* = 2 or 3 WMSS samples was provided by each participant). The orange horizontal line indicates the F^-^ concentration value estimated from its original product content. TFA, trifluoroacetate internal standard -CF_3_ group resonance.

High-field ^19^F NMR analysis was employed to explore the “loading” of WMSS samples with F^−^ arising from their treatment with a diluted SDF product *in vitro*. For baseline data, Figure 5A shows a 564.72 ppm ^19^F NMR spectrum of a typical WMSS sample arising from a typical “fasting” fluoride-containing WMSS sample collected from a healthy human participant; although not visible in this region of the ^19^F NMR spectral profile shown, this sample also contained TFA as an internal standard (*δ_F_* = −75.3 ppm) added following sample collection and preparation at a final concentration of 1.08 mmol/L. The F^−^ resonance clearly detectable in this spectrum had a signal-to-noise (STN) ratio of ca. 50, and values lower than several µmol/L were detectable and quantifiable in WMSSs using this technique. This sample had an F^−^ level of 16.4 µmol/L, a value somewhat lower than that of its mean baseline salivary concentration of 21.6 ± 20.0 µmol/L (0.41 ± 0.38 ppm) (24). Fluoride anion was indeed monitorable in most WMSS samples collected for analysis, but it should be noted that it was undetectable in several of those analysed.

The major technical hurdle for this successful trace level ^19^F NMR analysis was circumvention of interfering very broad, fast-relaxing ^19^F signals derived from a solid fluoropolymer material located within the NMR magnet probe-head. Nevertheless, our approach involved the removal of data points from the initiation of the free induction delay (FID), which contributed towards these very broad resonances, prior to the employment of a portion of this FID to allow prediction and replacement of data removed. This strategy gave rise to a flatter baseline, with only the narrow F^−^ and TFA internal standard resonances remaining unmodified. This FID processing technique was found to be very insightful, and both mathematically powerful and highly reproducible.

This approach was then extended to determine the fluoride content of saliva samples treated with a diluted sample of the commercially-available SDF product evaluated here, although the high level of sensitivity mandatory for F^−^ determinations in untreated samples was not required in this case. Moreover, it was also employed to directly determine the F^−^ anion content of the commercial SDF product employed for these studies.

^19^F NMR analysis of the SDF product alone revealed that although its Ag(I) and F^−^ content should have both been 2.76–3.15 mol/L for a 35%–40% (w/v) solution of it, as specified by the manufacturer (equivalent to 52,440–59,850 ppm), the level of F^−^ determined here by ^19^F NMR analysis was excessively higher than this value at 3.63 mol/L (68,941 ppm), i.e., an increase of approximately 23% over that commercially-specified. This observation is consistent with previous observations reported (29, 30). Figure 5B shows a typical ^19^F NMR spectrum acquired for determination of the F^−^ content of diluted SDF product solutions using the TFA internal standard.

Subsequently, fasting WMSS samples collected and prepared from *n* = 5 different healthy human participants were treated with an aliquot of a 1/10 diluted SDF product solution, and again this ^19^F NMR technique was employed for F^−^ determinations in this biofluid (mainly *n* = 3 different replicate samples collected from each volunteer) following removal of the Ag/AgCl deposit arising from the reaction of [Ag(NH_3_)_2_]^+^ with chloride and further salivary biomolecules; Figures 5C,D show typical ^19^F spectra acquired on a WMSS sample prior and subsequent to the addition of a diluted SDF clinical product solution respectively, as specified. For these samples, the predicted added F^−^ concentration was 4.58 mmol/L. Figure 2E shows a plot of mean ± 95% confidence intervals (CIs) for the ^19^F NMR-determined fluoride levels of replicate determinations for each of the separate samples evaluated (the baseline mean F^−^ level in untreated saliva samples was <20 µmol/L, and therefore its contribution towards the final F^−^ level was negligible). A completely randomized design, one-way ANOVA performed confirmed that there were no significant “between-participant” differences found, with the exception of one of them (sample A) having a significantly lower F^−^ level than that of sample A. This may be attributable to the uptake of this anion by the solid-phase Ag/AgCl nanoparticulate deposit formed in this sample, but not in the others. Indeed, one of the samples subjected to SEM analysis was found to contain traces of fluoride anion (Section 3.2.2).

In an additional experiment, we monitored the exact chemical shift and Δv_1/2_ values of the F^−^^19^F NMR resonance in *n* = 7 samples of WMSS in order to determine the influence of this biofluid medium and biomolecules therein on these important parameters. The ^19^F NMR dataset acquired was then analysed using a completely randomised design (one-way) type 1 analysis-of-variance (ANOVA) model, and this demonstrated that although there were no significant “between-participant” differences in *δ* values found (range of mean values −119.41 to −119.33 ppm only, *p* = 0.071), that between Δv_1/2_ was very highly significant indeed, with *p* = 6.02 × 10^−10^.

Mean±‘Between-Replicate Sample' SD Δv_1/2_ values for these seven participants were 6.32 ± 0.65, 6.74 ± 0.68, 6.53 ± 0.44, 8.43 ± 0.36, 10.26 ± 0.65, 10.95 ± 0.28 and 13.13 ± 0.36 Hz. Since the three lowest mean values observed are not much greater than those found in authentic samples of SDF in aqueous solution (Figure 1), these data indicate that for these samples, little or no interaction of F^−^ with exchangeable salivary metal ions [e.g., Ca^2+^ or Mg^2+^, or any residual solution-phase Ag(I) following its dominant precipitation as AgCl prior to Ag/AgCl CSNP generation] occurs. However, for those WMSS samples containing ^19^F signals with higher Δv_1/2_ values (i.e., >10 Hz), this may indicate that such interactions do indeed occur in these biofluids, but not for those samples yielding significantly lower values of this parameter (ca. 5 Hz).

#### 3.2.4 ^1^H NMR determination of NH_3_ in the clinically-applicable SDF product and WMSS samples treated with this agent

Similarly, changes in the ammonia content of WMSS samples were also monitored in order to evaluate its release and hence availability from an added SDF source following AgCl precipitation and/or ligand-exchange reactions with selected biomolecules. For this purpose, we employed our recently-developed ^1^H NMR method for its determination in this biofluid supernatant as ammonium ion (NH_4_^+^). However, since this method requires the prior acidification of samples to a pH value of 2.00, a process which would also be expected to liberate the NH_3_ ligand from SDF as NH_4_^+^, this approach may only be employed to check for the total NH_3_ level available before and after SDF addition to WMSS samples, and unfortunately not the amount of this ligand remaining complexed to any Ag(I) remaining following AgCl precipitation and ligand-exchange reactions.

The NH_3_ content of this product was specified by the manufacturer as 15%–20% (w/v), i.e., 8.82–11.76 mol/L. Baseline WMSS sample NH_4_^+^ concentrations were found to be 3.51, 4.59 and 5.83 mmol/L in these samples collected from different participants [values within the expected range of those previously reported (21)]. Following the addition of 10.0 µl of a 1/10-diluted solution of the SDF product to 0.70 ml of each of the *n* = 3 WMSS samples, however, these were elevated to 13.13, 13.28 and 14.36 mmol/L respectively, data yielding a mean increase in salivary NH_4_^+^ concentration of 8.95 ± 0.59 mmol/L (mean ± SD), which was within the specified 8.82–11.76 mol/L range predicted from the SDF product's NH_3_ contents noted above. Hence, data acquired from our SDF-WMSS experiments estimated an excess level of 49.6%–66.1% of added NH_3_ over that predicted from a consideration of the required two moles of this ligand per mole of Ag(I), although this range covers the manufacturer's 63% excess value.

Since NH_3_ is very soluble in chloroform, we elected to extract the excess of this ligand present in clinical SDF formulations and conduct preliminary ^15^N NMR analysis of those obtained. These experiments served to confirm that clear ^15^NH_3_ signals were observed in ^15^N spectra acquired on all three SDF products analysed, but not so for the SF products, including those for non-clinical uses, as expected (data not shown). Hence, assuming that no Ag(I)-coordinated NH_3_ was extractable into the chloroform medium, these data confirm that excess “free”, non-strongly-coordinated or perhaps even H-bonded NH_3_, was indeed present in these samples. These ^15^N NMR data will be reported in detail elsewhere.

Such results represent a major cause for concern in view of the foul odour of NH_3_, along with its potential toxic effects. It is very unlikely that co-ordinated NH_3_, as in [Ag(NH_3_)_2_]^+^, has a malodour similar to NH_3_ itself, and hence it is recommended that further caution should be taken by its manufacturer in order to circumvent this excessive added ligand issue during its production.

#### 3.2.5 FTIR-ATR analysis of human WMSS samples prior and subsequent to treatment with SDF, and Ag/AgCl nanoparticulate deposits derived therefrom

**Figure 6:**
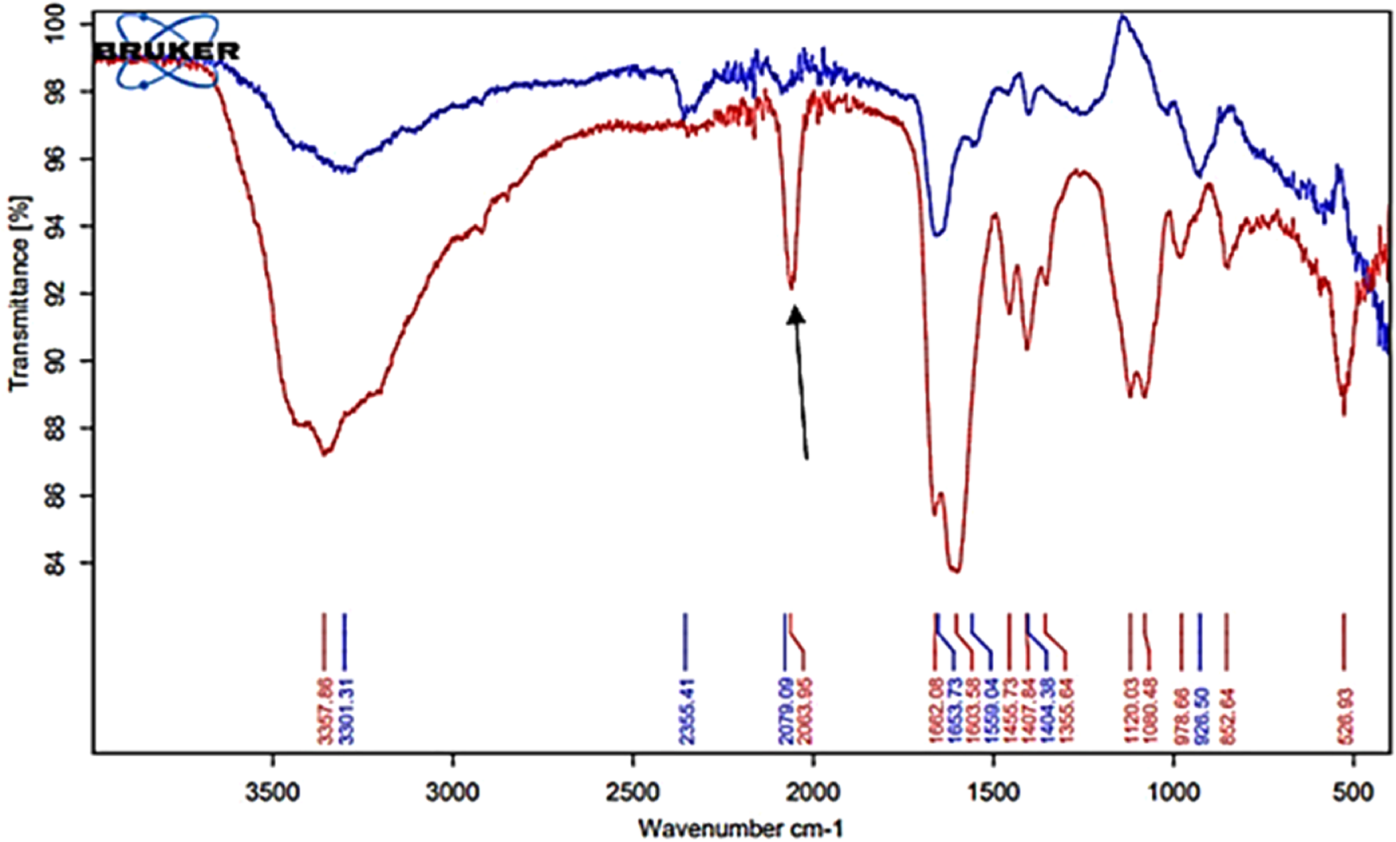
FTIR-ATR spectral profiles acquired on dried thin films of a control (untreated) WMSS sample (red spectrum) and that of the clear supernatant obtained following the sequential addition of SDF to these samples and removal of the Ag/AgCl-based nanoparticulate deposit formed (blue spectrum). The arrow indicates the thiocyanate anion C-N stretching band.

Figure 6 shows the FTIR-ATR spectra of a typical WMSS specimen (red spectrum) collected from a healthy control participant, and this contains a range of diverse absorption bands arising from different biomolecules and ions therein, including those assignable to proteins, lipids, carbohydrates and nucleic acids, along with thiocyanate anion. Indeed, this spectrum displays previously reported characteristic absorption bands visible for human saliva (31–33), such as those for proteins at 1,662 cm^−1^ (Amide I band, C=O stretching), 1,604 cm^−1^ (Amide II band, N–H bending), and 1,245 cm^−1^ (Amide III band). Moreover, within the spectral region for nucleic acids (1,100–850 cm^−1^), P=O asymmetrical and symmetrical stretching vibrations from the PO_2_ phosphodiester groups of phosphorylated biomolecules (1,080 cm^−1^) were also notable (although the 1,240 cm^−1^ band was obscured), as were the C–O stretching and linked C–O bending vibrations of the C–OH functional groups of carbohydrates located at 1,043 and 1,000 cm^−1^ (these including glycogen, but in the >8 h oral abstention period regimen required for the saliva sampling protocol of this study (Section 2.3), the majority of these detectable will presumably be attributable to those naturally present in fasted saliva specimens, including N-acetylated glycoproteins). Indeed, the molecularly-mobile carbohydrate side-chains of N-acetylated glycoproteins, and glycosylated proteins in general such as α-amylase, have absorption maxima within the 1,080–950 cm^−1^ spectral region (31–33), and in this study it was found that absorption bands centred at 977 and 954 cm^−1^ may be assignable to this enzyme, the latter being a shoulder only. Of some importance, also notable was a quite sharp band ascribable to the C-N stretch of thiocyanate anion (SCN^−^) at 2,064 cm^−1^, along with those within the higher frequency regions which are ascribable to lipids fatty acids, proteins, primary and secondary amines, and ammonium ion.

Additionally, the band located at 977 cm^−1^ may also be partially assignable to the DNA backbone stretching vibration, although again it should be noted that our preparation of WMSS samples involved the prior removal of cells, microbes and debris from the samples collected via a centrifugation step, as described in Section 2.3, and therefore it is likely that much of the DNA had been removed from these samples during this sample preparation step required.

The blue spectrum shown in Figure 6 corresponds to that of a thin film of a WMSS specimen obtained following added SDF-induced precipitation, and the removal of this deposit via centrifugation; this was derived from the same intact control WMSS sample giving rise to the red spectrum shown in this Figure. Clearly, there is a marked depletion of FTIR-ATR absorption bands in the blue spectrum, and this observation was reproducible for all WMSS samples subjected to this bioanalytical protocol. Indeed, the WMSS bands located at 527, 853, 979, 1,080, 1,120, 1,408, 1,456, 1,604, and 1,662 cm^−1^, together with all those within the 2,600–3,700 cm^−1^ spectral region (notably that at 3,358 cm^−1^), were virtually all removed from spectra acquired following Ag/AgCl nanoparticulate generation and its laboratory removal via centrifugation. Moreover, a major loss of SCN^−^ (clear band centred at 2,064 cm^−1^) from this biofluid was observed. As expected, the intensity of these absorption bands in FTIR-ATR spectra acquired on all the above SDF-treated, biomolecule-deplete WMSS samples were markedly lower than those of their untreated control counterparts.

**Figure 7:**
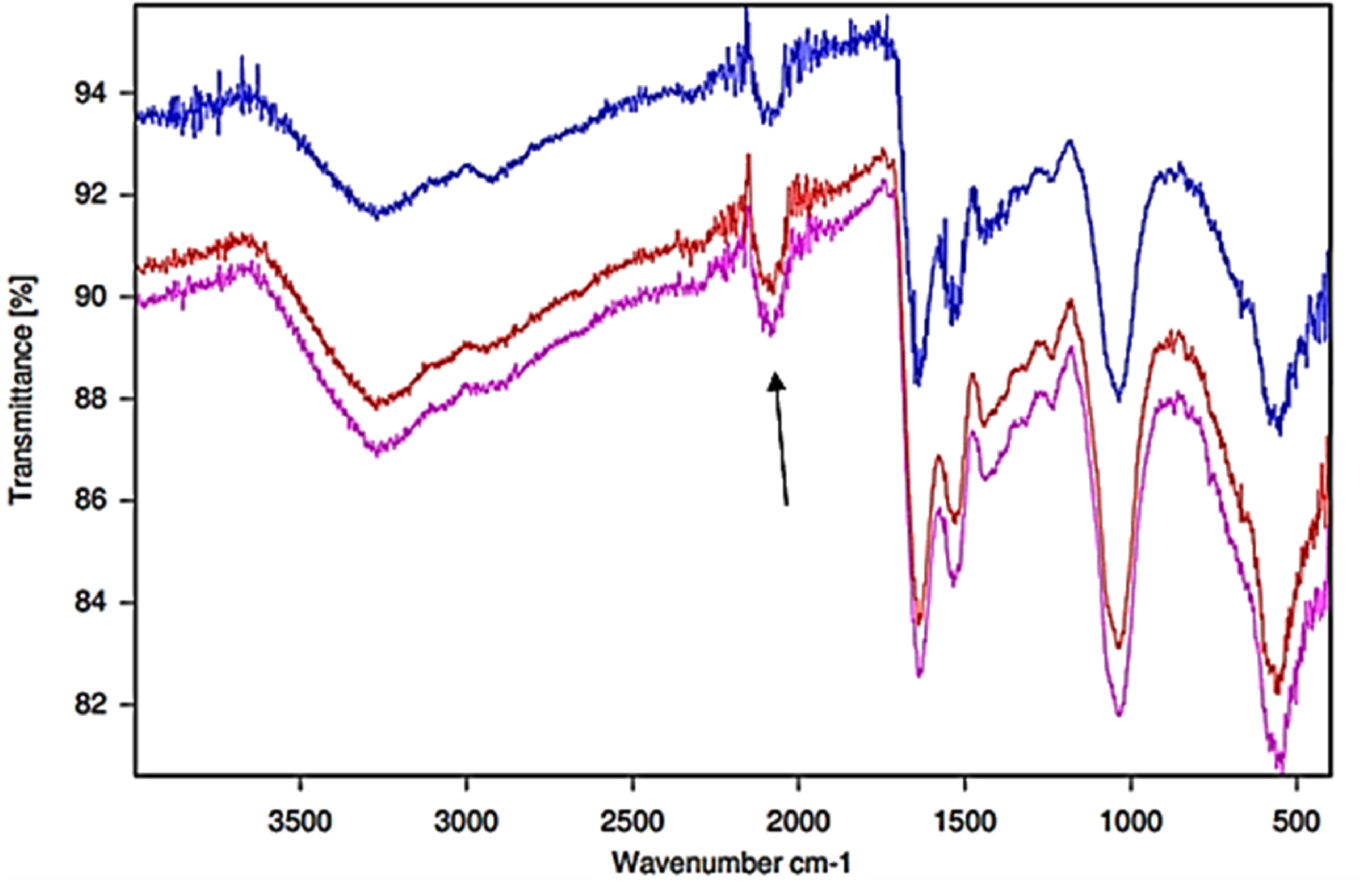
FTIR-ATR spectra of Ag/AgCl nanoparticulate solid deposits formed from the reactions of SDF added to human WMSS samples (*n* = 3 replicate samples collected from the same participant). The arrow indicates the thiocyanate anion C-N stretching band.

Hence, these observations were all consistent with the uptake of quite substantial amounts of a wide range of WMSS biomolecules by Ag/AgCl precipitates, a process which is slowly followed by the formation of biomolecule-induced and -protected nanoparticulate deposits subsequent to the addition of a final level of only 3.65 mmol/L of Ag(I) as SDF [concentration calculated from the manufacturer's specified SDF concentration of 2.95 mol/L, assuming a basal product Ag(I)-fluoride concentration of 37.5% (w/v)]. Also particularly notable was the quite high content of CO_2_ present in the residual WMSS samples (FTIR absorption band at 2,355 cm^−1^, blue spectrum in Figure 6). Hence, this agent may have infiltrated the samples analysed from the laboratory atmosphere during their periods of storage, or drying, for FTIR-ATR analysis, although this observation may also indicate that the nanoparticulates generated may contain some insoluble silver(I)-carbonate, an observation consistent with the FTIR absorption band located at 558 cm^−1^ (Table 1 and Figure 7), which has been tentatively assigned to a metal ion-ligand Ag(I)-O (34) and/or Ag(I)-Cl and/or Ag(I)-N stretching vibration(s), in addition to the significant amounts of both carbon and oxygen found therein by SEM analysis (Figure 3). However, if it is assignable to an Ag(I)-O stretch, this band may also arise from Ag(I)-phosphate species therein.

**Table 1:** Tabulated list of maximum absorption frequencies of bands present in the FTIR-ATR spectral profiles acquired on thin films of control (untreated) WMSS samples (coded 1-20, denoted in black), along with those detectable in corresponding spectra of the dried Ag/AgCl nanoparticulate deposits formed from the addition of SDF to these samples (coded A-P, in blue).

Band code	Absorption band frequency (cm^−1^)	Biomolecular assignment(s)
1	527	v(OCN) (tentative)
A	558	Ag(I)-O or Ag(I)-Cl stretch (tentative)
2	853	H-N-H rocking/left DNA helix, z-form (tentative)
3	924 (sh)	Membrane lipids (phospholipids)/carbohydrates ν(PO_2_^−^) inorganic phosphates δ(COH), δ(COC) carbohydrates νas(CH_3_–N) phospholipids
4	954 (sh)	α-Amylase (tentative)/glucose/glycogen/sugar moieties (C-C and C-O stretches)
5	977	vs. (C-N-C) proteins/α-Amylase (tentative)/DNA backbone (tentative) ν(PO_2_^−^) inorganic phosphates
6	1,000 (sh)	Glycogen (C-O stretch)/polysaccharides
B	1,043	α-amylase/glucose, glycogen and sugar residues (C-C and C-O stretches) ν(CO), ρb(C–O–H) glycosylated α-amylase, mucins or alternative sugar residues
C	1,078	vas(C-N-C) sugar residues, glycosylated proteins, proteins and phosphate compounds/phospholipids vs.(C-O-C stretch)/vs.(C-O) stretch νs(PO_2_^−^) inorganic phosphates νs(PO_2_^−^), νas(PO_2_^−^) phosphate group of phospholipids ν(CO), ρb(C–O–H) glycosylated α-amylase, mucins or other sugar residues
7	1,080	vas(C-N-C) sugar moieties/glycosylated proteins/proteins/phosphate compounds/phospholipids vs. (C-O-C stretch)/vs.(C-O) stretch νs(PO_2_^−^) inorganic phosphates νs(PO_2_^−^), νas(PO_2_^−^) phosphate group of phospholipids ν(CO), ρb(C–O–H) glycosylated α-amylase, mucins or other sugar residues
8	1,120 (sh)	vas(nucleic acids)/phospholipids (vas(PO_4_))/carbohydrates ν(C–O), ν(C–O–C) νas(PO_2_^−^) Inorganic phosphates ν(CO), ν(C–O–H) serine, threonine and tyrosine residues of proteins/carbohydrates
D	1,234 (sh)	Nucleic acids/phospholipids (vas(PO_4_)/amide III/phospholipids νas(PO_2_^−^) inorganic phosphates
E	1,240	Amide III/assymetric and symmetric stretch PO_4_/Ester bond/phospholipids (ν(C–N) of proteins in α-helix conformation)
9	1,245 (sh)	Amide III/assymetric and symmetric stretch PO_4_/Ester bond/phospholipids (ν(C–N) of proteins in α-helix conformation) νas(PO_2_^−^) phosphate group of phospholipids νas(PO_2_^−^) inorganic phosphates
F	1,324 (sh)	Amide III/N-H bending ν(CN), ρb(NH) Amide III (α-amylase, albumin, mucins and proline-rich proteins, SIgA)
10	1,356	Amide III/N-H bending ρb(CH_2_) phospholipids, fatty acids, triacylglycerols and amino acid side-chains
G	1,385	Proteins vs.(CH_3_)
11	1,408	Proteins vs.(CH_3_) νs(CO_2_^−^) fatty acids ρb(CH_3_) proteins
12	1,456	δ(N-H) proteins/vas(CH_3_)lipids
H	1,527	Amide II band (N-H bending/C-N and ν(C-C) proteins, tyrosine) Tyrosine-containing proteins (α-amylase, albumin, mucins, proline-rich proteins and SIgA) ρb(NH), ν(C = N), ν(C = C)
13	1,604	Amide II band
I	1,638	Amide I band (C = O stretching/C = O of carboxylates/carboxylic acids or esters/α-helix/C = N stretching) ν(C = O) Amide I: unordered structure ν(C = O)/ν(C = C) Amide I: β-sheet structure ν(C = C) Amide I: β-sheet structure ν(C = O), δ(CN), δ(NH) amide I: α-helix
14	1,662	Amide I band, C = O stretching/C = O of carboxylates/carboxylic acids or esters/α-helix/C = N stretching ν(C = O) Amide I: unordered structure ν(C = O), δ(CN), δ(NH) amide I: α-helix νs(C = O) Amide I: disordered structure-solvated ν(C = O) Amide I: unordered random coils and turns ν(C = O), ν(CN), ρb(NH) Amide I: anti-parallel β-sheet
15	2,064	SCN^−^ (C-N stretch)
J	2,105	SCN^−^ (C-N stretch)
K	2,334	Possible S-H stretch (L-cysteine, free or protein residue)
16	2,340	CO_2_ *vas*(O = C = O)
L	2,355	CO_2_ *vas*(O = C = O)
17	2,865 (sh)	νs(CH_3_) lipids/protein side-chains v(C-H)/NH_4_^+^ νs(CH_2_) lipids (cholesterol and mono-/diacylglycerols)
18	2,937 (sh)	vas(CH_2_) lipids/fatty acids
M	2,961 (sh)	vas(CH_2_) lipids/fatty acids/protein side-chains
N	3,099 (sh)	N-H stretch *v*(NH_3_)/*v*(C-H) amide B/ring (α-amylase, albumin, mucins, proline-rich proteins, SIgA)
19	3,209 (sh)	ρb(N-H) and/or ρb(O-H) bend
O	3,305	Amide A vas(N-H): nucleic acids/primary and secondary amines
20	3,358 (sh)	Amide A vas(N-H): nucleic acids/primary and secondary amines
P	3,441 (sh)	Amide A: primary and secondary amines

Biomolecular assignments are provided in the final column. sh, shoulder; v, vas, vs. refer to stretching, asymmetric and symmetric vibrations; δ, deformation vibration; δas, asymmetric deformation vibration; ρb, bending vibration; SIgA, immunoglobulin A.

Table 1 provides a full list of absorption frequency maxima of bands present in the FTIR-ATR spectra of thin films of control (untreated) WMSS samples, together with those found in corresponding spectra of the dried Ag/AgCl nanoparticulate deposits resulting from their treatment with SDF according to Section 2.3. A full range of known or possible biomolecular assignments are provided, although as expected, there was a significant level of superimposition of their individual contributions towards each selected frequency band.

Moreover, Figure 7 displays the corresponding FTIR-ATR spectra of the Ag/AgCl deposit formed following the addition of SDF to replicate WMSS samples, and similarities and differences between these spectral profiles and those of the untreated control samples are tabulated in Table 1. Notably, it was possible to distinguish between these two types of profiles on the basis of differences in absorbance and functional group band wavenumber displacements between them, which represent major modifications in their biomolecular compositions. Indeed, for the dry thin film of the deposited nanoparticulate matrix, an absorption band located at 3,358 cm^−1^ in the untreated WMSS samples (predominantly Amide A v(N-H) assignment) was shifted to 3,305 cm^−1^ (bands 20 and O respectively); that at 2,937 cm^−1^ [lipids/fatty acids (vas CH_2_)] was shifted to higher frequency (2,961 cm^−1^) (bands 18 and M respectively); the characteristic CO_2_ and thiocyanate anion bands located at 2,340 and 2,064 cm^−1^, respectively, were displaced to 2,345 and 2,105 cm^−1^, respectively (bands 16 and L respectively for CO_2_/bands 15 and J respectively for SCN^−^); the Amide I C = O stretch at 1,662 cm^−1^ was shifted to 1,638 cm^−1^ (bands 16 and I respectively); the Amide II band (1,604 cm^−1^) appeared to be shifted to 1,527 cm^−1^ (bands 13 and H respectively); the protein vs. (CH_3_) band at 1,408 cm^−1^ was shifted to 1,385 cm^−1^ (bands 11 and G respectively); the Amide II N-H bending band was displaced from 1,356 to 1,324 cm^−1^ (bands 10 and F respectively); the Amide III/asymmetric and symmetric PO_4_ stretch band at 1,240 cm^−1^ was shifted to 1,245 cm^−1^ (bands 9 and E respectively); that at 1,120 cm^−1^ (nucleic acids, phospholipids and carbohydrates) was shifted to higher frequency (1,234 cm^−1^) (bands 8 and D respectively); and the band at 1,080 cm^−1^ (assigned to sugar moieties/glycosylated proteins/proteins/PO_4_ compounds/phospholipids (C-O-C stretch)/C-O stretch) was displaced to slightly lower frequency (1,078 cm^−1^) (bands 7 and C respectively). Information available in Table 1 revealed that the absorption band assigned to the nanoparticulate surface-adsorbed SCN^−^ had a frequency value which was approximately 40 cm^−1^ higher than that of the “free” species present in the untreated WMSS samples, and this may indicate its complexation of Ag(I).

Consistent with the detection of phosphate stretching vibrations in the FTIR-ATR spectra of the dried nanoparticulate deposits generated, qualitative pilot ^31^P NMR analysis of acidic (pH 2.00) extracts of these confirmed the presence of detectable levels of inorganic phosphates therein, an observation that they may be available for the complexation or part-complexation of Ag(I) ions therein. Inorganic phosphate was also readily detectable in WMSS samples, as expected (data not shown).

These Ag/AgCl nanoparticle absorption bands mainly correspond to protein and amino acid amide C=O, C–N, C–H, C–C, O–H, N–H, phosphate, lipids/fatty acids and perhaps nucleic acid groups on the nanoparticle surface, one or more of which may have been responsible for the reduction of Ag(I) to Ag(0). However, the FTIR-ATM spectra acquired have confirmed that there are also significant levels of SCN^−^ and CO_2_ detectable therein, almost all of the former being transferred from the WMSS samples themselves so that there were only limited quantities of this anion remaining in the WMSS samples following SDF treatment and centrifugation (Figure 6). The vibrational bond frequencies located between 3,800 and 2,200 cm^−1^ of dried Ag/AgCl nanoparticulate samples largely represent surface-adsorbed O–H, N–H, C–N C–C and C–H bond stretching absorption bands of a range of biomolecular species, including SCN^−^ and the asymmetric C=O stretch of adsorbed CO_2_.

## 4 Discussion

### 4.1 Overview of bioanalytical results acquired

Our intensive laboratory-based studies reported here have successfully demonstrated that:

(1) Commercially-available SF, but not SDF, product solutions for clinical use contain not just singular, isolated and/or aquated Ag(I) and F^−^ ions, but a SF complex containing rapidly-exchanging F^−^ at the Ag(I) metal centre, an observation which may indeed serve to significantly influence the reactivities of both ion species on application *in vivo*, and hence its MoAs, including its potential ability to divert delivery of F^−^ to hydroxyapatite for clinical hard tissue strengthening purposes. Moreover, our dissolution/dilution kinetic experiments described in Section 3.1 clearly confirm a structural heterogeneity of SF present in its clinical product solutions, and it certainly appears that there is a major structural change occurring on dilution of this medium, with the *δ_F_* and Δv_1/2_ values of its F^−^ resonance displaying major time-dependent decreases up to time-points of >6 h at ambient temperature. These data are also fully consistent with the studies of Higashijima et al. (35), who found that the F^−^ resonance in ^19^F NMR spectra of simple aqueous model fluoride solutions underwent a substantial level of broadening in the presence of added Mg^2+^ ions. This phenomenon was attributed to the formation of a solution-phase ion-pair complex in that study, but this broadening is also ascribable to a fast exchange process at the metal ion centre on the NMR timescale. The authors of the current study have repeated this experiment in aqueous solution with differing added Mg^2+^ and F^−^ concentrations, and can confirm that the Mg^2+^-mediated broadening of the F^−^ signal was indeed observed.
(2) For our studies with SF, the F_2_H^−^ species may also be featured as an exchangeable ligand, most notably at acidic or mildly acidic pH values. However, this potential Ag(I) ligand has an ^19^F chemical shift value of −153 ppm when present in the “free” state in aqueous solutions containing 10% ^2^H_2_O (Grootveld et al., unpublished data, Figure 1D and 18), and is formed from the reaction (solvation) of F^−^ with HF (pKa value 3.2). It is also likely that acidic SF products which notably contain excess levels of F^−^ (30) also contain Ag(I)-complexed and/or free F_2_H^−^ species, which may also exert significant bioactive properties and possible favourable therapeutic effects. Evidence available indicates that the Ag(I)-complexed form is SF solvated with one molar equivalent of hydrogen fluoride (HF). Moreover, this HF solvation process appears to effectively prevent water solvation at the Ag(I) centre, and thus protects it against H_2_O- or OH^−^-mediated ligand-substitution and hydrolysis, which are sequentially followed by the deposition of its solid Ag_2_O and Ag(0) degradation products at neutral or near-neutral pH values.

Since the pKa value of HF is only 3.2, at a pH value of 5.0, HF will be present at a concentration of 46.8 mmol/L for a typical 3.00 mol/L solution of F^−^ (when taking into account the total F^−^ present), and hence it is anticipated that significant levels of [Ag(I)-F_2_H] will form at this or even less acidotic environments.

Potentially, this estimated HF content is of some toxicological significance, and this should represent a concern for those engaged in the commercial production and clinical application of such acidulated SF products. The pH value of 5.0 used above is actually higher than that estimated for primary root carious dentin lesions [ca. 4.5 (36)], and therefore greater amounts of this toxin will be expected to be generated at these sites. Worryingly, at such low pH values, solution-state or even gaseous hydrogen fluoride (HF) may be liberated from the aqueous solution medium, and hence steps should be taken to circumvent this in view of its contact with or inhalation by human patients. Indeed, severe burns are known to be caused by contact with aqueous HF, and toxic effects arising from the acute inhalation of its gaseous form have been explored in different laboratory animal model systems. Additionally, the irritant effects of HF have been investigated, and it has also been found to act as a severe irritant towards the nasal passages, eyes and skin (37).

However, if any SF remains at its clinical application site following therapy, then in principle it may serve to protect against the toxicological effects exerted by HF, via a mechanism involving the scavenging of F^−^ solvated with this acid (as HF_2_^−^) in the form of a [Ag(I)-F_2_H] complex. However, this complex is known to etch borosilicate glassware (23), so how safe will tooth enamel be when exposed to this agent?

(3) The commercial batch of one of the SDF solution products employed for these studies contains and delivers to WMSS samples excessive levels of both F^−^ and NH_3_ (23 and 63 molar % respectively), that of the former counter-ion present confirming previous observations made by other researchers, e.g., (29, 30), who found that both the fluoride and silver(I) contents of a series of a series of commercially-available clinical SDF products deviated markedly from their specified values. However, the excess NH_3_ ligand present was actually specified by the manufacturer to be within our ^1^H NMR-estimated concentration range, i.e., 50%–66% higher than that required for satisfaction of the 2:1 NH_3_:Ag(I) stoichiometry of [Ag(I)(NH_3_)_2_]^+^. Of notable interest, the manufacturer-specified product range provided is substantially higher than that needed to satisfy the 2.0 molar equivalents of it required to form the [Ag(NH_3_)_2_]^+^ ion (which should, in principle, be equivalent to exactly twice the range provided for Ag(-I)-fluoride, i.e., 5.52–6.30 mol/L), and this confirmed that the SDF product evaluated here certainly contained a large excess of the NH_3_ ligand added by the manufacturer, specifically an average molar excess of 63% over that required to satisfy the above 2:1 stoichiometry when estimated from our experiments, assuming a mean Ag(I)-fluoride product content of 37.5% (w/v) (equivalent to 2.95 mmol/L).
(4) SDF primarily forms off-white precipitates on addition to WMSS samples, from which develop light exposure–dependent chromophoric nanoparticulate species in a time-dependent manner, these containing high contents of Ag and Cl (with an approximate Ag: Cl ratio of 1.25:1), and which appear to be encapsulated by a range of salivary biomolecules, including proteins, carboxylates, amino acids and perhaps DNA (these biomolecules may also serve to cap and stabilise these nanoparticles, in addition to their roles played in reducing Ag(I) to Ag(0)). Salivary reducing agents available for the conversion of Ag(I) to metallic Ag (Ag(0)) encompass L-cysteine [total mean salivary thiol level approximately 40 µmol/L in healthy individuals (38)], both low-molecular-mass and protein-incorporated, thiocyanate anion (SCN^−^), which is present in this biofluid at quite high mmol/L levels, although it is higher in tobacco smokers since it arises from the metabolism of cigarette smoke hydrogen cyanide (39), and the phenolic amino acid L-tyrosine (approximately 10 µmol/L) (40), along with any residual dietary reductants such as ergothioneine, ascorbate and further phenolics (presumably very limited concentrations of the latter only, however, in view of the lengthy oral abstention episode required here for participant whole mouth saliva sample provision). Similar results were obtained with SF and Ag(I)-nitrate addition to WMSS samples.
(5) FTIR-ATR analysis revealed that salivary thiocyanate anion (SCN^−^), an essential precursor to the highly microbicidal hypothiocyanite anion (OSCN^−^), which additionally serves in the peroxidase enzyme system by counteracting the toxicity of hydrogen peroxide generated by oral bacteria (41), is also transferred onto the Ag/AgCl precipitates and nanoparticules formed from the interaction of SDF with WMSSs, and hence insoluble [Ag(I)-SCN] may also form part of this depositional/nanoparticulate matrix. It is also likely that in view of the relatively high levels of F^−^ added to WMSS samples as SDF, the deposit primarily formed, and nanoparticulates generated therefrom subsequently, may also contain some precipitated calcium- or magnesium-fluorides). Indeed, salivary concentration ranges of these metal ions have been reported to be 0.87–1.33 and 0.70–1.67 mmol/L respectively (42). Furthermore inorganic phosphate levels in unstimulated whole human saliva samples were found to range from 2.0 to 12.6 mmol/L, and from this it seems likely some of the silver(I) present in these deposits is in the form of one or more insoluble silver(I)-phosphate complexes (43).

Although it appears that most, if not all Ag(I) ions added to our WMSS samples as SDF were consumed via its primary precipitation as AgCl from reaction with salivary chloride, from the Δv_1/2_ determinations made on the ^19^F NMR resonances of different SDF-treated WMSS samples, it is possible that for at least some of them, sufficient Ag(I)-F species with exchange-broadened ^19^F resonances are generated therein following the release of Ag(I)-coordinated NH_3_ via ligand-exchange reactions on SDF addition, and further experiments to explore this are planned by the authors. However, it is also possible that the F^−^ signal broadenings observed in some samples, but not all, arise from the interactions of salivary Ca^2+^ or Mg^2+^ ions with this anion, the signal observed representing the biofluid-soluble portions of highly- or less-insoluble CaF_2_ or MgF_2­_ respectively, as has been previously observed for the latter in the study reported in Ref (35). However, this broadening effect may also be explicable by its binding to and/or exchange with salivary proteins or alternative macromolecules.

If any solid-state CaF_2_ is generated, then it is likely to be present in the precipitated Ag/AgCl CSNP samples analysed. However, only low amounts of Ca^2+^, and even less Mg^2+^, were found to be present therein, (mean atomic composition levels of only 1.5% and 0.2% respectively) using SEM analysis. Likewise, fluoride was only marginally detectable at a mean content value of 0.3%, as was phosphate at a level of only 0.6%. It should also be noted that the solubilities of CaF_2_ and MgF_2_ in water are only 0.20 and 1.22 mmol/L respectively (values equivalent to 0.40 and 2.44 mmol/L F^−^ respectively).

The transfer of Ca^2+^ ions in CaF_2_ to inorganic phosphate present in human saliva also remains a possibility, although the atomic content of phosphorous (presumably virtually all as inorganic or organic phosphates) in the CSNP samples analysed by SEM analysis is very low. Notwithstanding, a low wavenumber absorption band observed in FTIR-ATR spectra acquired on the solid material isolated may be ascribable to an Ag(I)-O or Ag(I)-Cl stretch (Table 1). Hence, if the former, this band could arise from silver(I)-phosphate present therein. Consistent with this, absorption bands assignable to a series of inorganic phosphates and phosphate function-containing biomolecules are detectable elsewhere in the FTIR-ATR spectra acquired.

(6) Ag/AgCl nanoparticulates are known to exert potent microbicidal and cariostatic properties (44–46). Notably, current advancements in nanotechnology have recently afforded major developments in the clinical use of nanomaterials, and developments with silver nanoparticles now offer alternative treatment options for clinical use in dental practices. In principle, such materials with optimised physicochemical properties may be employed for these purposes. In view of these bactericidal properties, the biofluid-catalysed *in vivo* “autoconstruction” of these nanoparticulates on exposure of these highly water-soluble, therapeutic silver(I) complexes to human saliva observed here may serve as a key mechanism for the favourable caries arrest properties of both SDF and SF. The authors have elected to represent this “*in vivo* salivary-catalysed autoconstruction of nanoparticulates” process by the acronym IV-SCAN. Perhaps SDF and SF therapies for the arrest of dental caries may only serve as precusorial “pro-metallodrugs” for the *in vivo* generation of therapeutically-active CSNPs? To the best of the authors’ knowledge, this is the very first time that ^19^F NMR analysis has been applied to monitor not only the composition and solution status of SDF and Ag(I)-fluoride products manufactured for the clinical arrest of dental caries, but also their biomolecular reactivities, and biomolecular fate of their constituents, in “real-life” human saliva specimens.

Many of the FTIR-ATR absorption bands observed at a range of vibrational frequencies in the spectral profiles of the WMSS-derived Ag/AgCl nanoparticles were ascribable to both high- and low-molecular-mass biomolecules present in this biofluid, and therefore these were not only responsible for their biosynthesis, but also potentially act as capping and stabilizing agents for these materials.

Although it appears that the majority of the Ag(I) cation added to this biofluid is consumed by its primary deposition as AgCl, followed by the time- and light-dependent development of CSNP species, that remaining may provide valuable information regarding its ability to form perhaps bioactive complexes with biomolecular ligands available therein, e.g., with salivary thiols such as cysteine [including the “target” cysteine residue in collagen (13, 14)], the tripeptide glutathione, and/or thiocyanate. However, using SEM, we were able to monitor the elemental constitution of the Ag/AgCl nanoparticulate deposits formed from the reactions of SDF with WMSS agents, particularly Ag(I) and chloride, but also with smaller quantities of FTIR-detected thiocyanate anion (Figure 7), amino acids and further biomolecules. Notably, the sulphur level of the nanoparticulate deposits formed was approximately 1.0 weight %, and presumably much of this is attributable to the SCN^−^ ligand.

The size (diameter) dimensions of the CSNPs generated in our IV-SCAN experiments appear to be somewhat higher than those commonly found in “green synthesis” generations, although those with particle sizes as high as 40–100 nm have been previously reported (47). Clearly, this enhanced diameter will exert a significant influence on the potential cariostatic and microbicidal, and hence overall therapeutic activities of these nanoparticles, and hence further studies are currently in progress to investigate this parameter and its potential effect on therapeutic activities.

Intriguingly, Motahhari et al. (48) found that a modified silver nanoparticle preparation could potentially be employed for the analytical determination of F^−^ anion, and this method relied on the ability of this analyte to anti-aggregate this nanoparticle preparation, a process which putatively involved the co-operative adsorption of fluoride ions onto the surface of silver nanoparticles, and a stripping of the sulphonate (-SO_3_^−^) function of the sulphanilic acid-catechol stabiliser employed. This disaggregation process was accompanied by a colour change of brown to yellow, and corresponded to absorbance increases and decreases at 397 and 508 nm respectively. Hence, the fluoride concentrations employed in our experiments involving the addition of diluted SDF solutions to WMSS samples, which are much lower than those used when SDF is clinically-applied, will be expected to exert major, perhaps disaggregating, effects when such Ag/AgCl nanoparticles are generated, both *in vitro* and *in vivo*.

Of particular interest to this study, our results regarding the accumulation of solid AgCl in dentin crevices and fissures, or porous demineralized enamel, have served to provide evidence for an alternative mechanism for the therapeutic activities of SDF and SF products, along with their deposition as dark/black metallic Ag-containing residues (Figure 3). Indeed, this accumulation within these sites, which are sheltered against tongue actions, is expected to be of sufficient duration for AgCl precipitates to develop CSNPs through biomolecular and/or photolytic reduction processes as a precusorial stage to the build-up of blackened metallic Ag(0) deposits at such locations, as opposed to the primary generation of insoluble Ag(I)-phosphate complexes proposed to arise from the reactions of Ag(I) with hydroxyapatite or alternative phosphate sources and species (5). Hence, the time-dependent shielding of CSNPs so produced may indeed promote any therapeutic caries-arresting effects that they may exert at this environment, events preceding their final decomposition to metallic Ag.

Consistent with the appearance of blackened metallic Ag(0) stains at active caries disease sites, this process is very unlikely to occur on the surface of smooth mineralized hard tissues since precipitated AgCl will simply be dislodged therefrom via tongue movement actions, and hence there will be insufficient time available for it to be sequentially converted to CSNPs and then deposited as dark-coloured Ag metal materials.

(7) Any insoluble CaF_2_ generated from the reaction of product F^−^ with endogenous Ca^2+^ may indeed serve to block dentinal tubules via their deposition therein, and therefore it may act as a desensitising agent in this context. Moreover, this process may also give rise to a bioenvironment which promotes the generation of further fluorohydroxyapatite for supplementation of the caries-arrest properties of SDF and SF products.

### 4.2 Access of human saliva to SDF and SF application sites during caries-arresting treatment

As fully documented in (49), the clinical application of SDF in the dental surgery requires (1) the removal of any gross debris from cavitations in order to permit improved access of the SDF drop medium to denatured enamel; (2) the isolation of all areas to be treated with cotton rolls or alternative segregation strategies; (3) the use of cotton rolls again, or other approaches, for the isolation of treatment site options (if materials for the coating protection of surrounding gingival tissues such as cocoa butter are applied, caution should be taken not to coat caries lesion surfaces); (4) that attentive care should be taken during the application of the SDF therapy on primary teeth which are adjacent to permanent anterior teeth that have or may have white spot (non-cavitated) lesions in order to circumvent unintentional black staining—the cautious use of a micro-brush is usually sufficient to overcome SDF's exposure to soft tissues, both intra- and extra-oral; and (5) that the lesion is dried with a moderate compressed air flow. SDF is then directly applied to the affected tooth surface, and care should be taken to remove excess volumes of it using cotton rolls or pellets, or gauze, in order to attenuate any systemic absorption. The application time is recommended to be at least one minute, although this is inevitably shorter for very young or intractable patients. A gentle application of compressed air is then used until the applied SDF has dried (isolation for a period of 3 min. is required during this stage of the treatment). Post-SDF treatment, the entire dentition may then be treated with 5% (w/w) sodium fluoride varnish, in order to avoid the further development of dental caries at all susceptible locations.

Hence, of all these steps, only the removal of gross debris, and isolation of the treatment area with cotton rolls or other media to prevent salivary moistening, may be considered to diminish the access of a salivary contaminant to this environment. However, in contrast, the air-drying phases, which are required both immediately before and after treatment application, could be considered to effectively concentrate salivary biomolecules at the SDF application site, if indeed there are any traces of this biofluid remaining thereon at these application points, and also even during the one minute treatment episode. Following its clinical application, SDF may continue to be exposed to this biofluid, although much of it will be converted to a series of biotransformation products at the later post-treatment stage, e.g., silver(I)-phosphate and insoluble dark brown-coloured silver(I) oxide for silver(I), metallic silver [Ag(0)] and fluorohydroxyapatite and calcium fluoride (CaF_2_) for the fluoride counter-ion, as discussed below. In principle, such biotransformation products will also be exposed to biomolecules present in human saliva. Moreover, if lipid-based cocoa butter is used as a protectant for adjoining gingival tissues, could any agents therein chemically react with SDF? Interestingly, unsaturated fatty acids are known to reversibly form polar complexes with silver(I) ions (50). In fact, this is the case for all unsaturated functions in organic biomolecules, in addition to fatty acids, and hence the water-soluble organic acid anion fumarate, which is ^1^H NMR-detectable in human saliva (20), may also act as a significant Ag(I) complexant in this biofluid.

The fact that the mean human salivary fluoride level was elevated 160-fold immediately after a recommended SDF application *in vivo* (51) has provided strong evidence that SDF does indeed come into contact with human saliva during its albeit short clinical treatment regimen. Furthermore, these values remained 1.8-fold greater than the baseline level even at the 1.0 h post-SDF treatment time-point.

### 4.3 Analytical monitoring of SDF and SF products for clinical use

The current study's experiments focused on the ^19^F and ^1^H NMR analysis of fluoride anion and ammonia, respectively, in a commercial SDF product for clinical use clearly demonstrated that it contained excessive levels of both these agents therein, specifically +23% and +63% of their expected values respectively; results obtained for F^−^are consistent with those made by other researchers in this area. Notably, independent analytical laboratory scrutiny has previously been applied to the silver(I) and fluoride anion contents of both SDF and SF products for clinical applications in dentistry. Indeed, in 2013 Mei et al. (29) investigated the short-term stability, what was described as “free” F^−^ levels, and pH values of three different commercial products (Cariestop, of both 12% and 30% (w/v) SDF content products, and the Saforide 38% (w/v) SDF product). These investigations found that the mean F^−^ contents of the freshly-opened products were 12,525 ± 450, 13,200 ± 2,060 and 55,800 ± 2,536 ppm, respectively, values which should have been 14,200, 35,400 and 44,800 ppm, respectively. Hence, there was a very large negative discrepancy in F^−^ anion content for the Cariestop 30% (w/v) product (−63%), and a large positive one for the Saforide formulation (+25%). Average pH values for these products were found to be 9.4 ± 0.1, 10.4 ± 0.1 and 10.2 ± 0.2, respectively. At both the 7- and 28-day storage time-points, no significant differences in product F^−^ levels, nor pH values, were detectable.

In a similar investigation performed in 2021, Patel et al. (52) monitored discrepancies between the “free” fluoride and silver ion concentrations of seven different commercial SDF product preparations, together with their pH values. Their results found very worrying deviations between the “correct”, predicted values and those arising from laboratory analysis. Indeed, one 30% (w/v) SDF product was found to be comprised of approximately only one-half the expected F^−^ anion composition, along with a much greater silver level than anticipated (expected values were 16,343 ppm F^−^ and 246,000 ppm Ag). For the remaining six products, which specified a 38% (w/v) SDF concentration, however, mean F^−^ and Ag contents were 74,802 and 326,000 ppm respectively (expected values were 44,800 and 253,870 ppm respectively). However, these products had very wide ranges for these values, with 36,457–120,760 ppm for F^−^, and 246,000–425,451 ppm for silver. Again, these values seem to deviate wildly from their expected values, and it would appear that the commercial organizations responsible for their manufacture had not efficiently engaged and maintained their quality control systems properly. The pH values of these solutions ranged from being at or close to neutrality (pH 7.0), to reasonably alkaline (pH 10.3).

A further laboratory-based investigation conducted by Gotjamanos and Afonso (30) found mean fluoride levels, which were substantially higher than the expected 60,000 ppm, for a 40% (w/v) neutral solution of SF. In view of these findings, the researchers involved erroneously suggested that some SF products contain silver(II)-fluoride (Ag(II)F_2_) rather than SF itself (the latter of which has an expected 1:1 [F^−^]:[Ag(I)] concentration ratio), along with hydrofluoric acid (HF). However, this remains highly speculative, since unlike Ag(I), Ag(II) cation acts as an extremely potent oxidising agent, and with the redox potential of the Ag(II)/Ag(I) couple being very close to +2.0 V, it may oxidise water to O_2_ and even ozone (O_3_), itself being converted back to Ag(I), or Ag(0) in the process. Hence, the presence of Ag(II)F_2_ in commercially-available aqueous SF formulations designed for oral healthcare applications can certainly be ruled out! Nevertheless, the authors of Ref (30). also conducted a risk of toxicity review, which indicated that the clinical use of this product had a toxicity risk which could give rise to dental fluorosis in young children.

A more recent study (53) explored the alkalinity and fluoride and silver ion contents of five commercially-available SDF products (specifically Advantage Arrest, e-SDF, Riva Star, Saforide, and Topamine) and hence their overall stability throughout a 180-day period of storage at 25°C; *n* = 6 bottles of each product were evaluated. Samples were visually examined and analysed on the 0, 30, 60, 90 and 180 day time-points, that at 0 representing the time corresponding to freshly-opened bottles. During this 180 day period, the pH values of these products did not alter significantly (these were 9.8–9.8, 10.5–10.6, 13.0–13.1, 9.8–9.8 and 9.3–9.4 for the above products, respectively). However, the fluoride and silver concentrations of these products decreased following the 60 day storage duration (>5% reductions were observed at this time). Moreover, the time taken for these products to form a black metallic silver precipitate when exposed to ambient room light (500 lx) at 25°C (to the nearest hourly integer) was 17, 12, 6, 7 and 7 h, respectively. However, there appears to be no correlation between pH values of these products and their precipitation times, although that with the shortest precipitation time (6 h) had the highest pH value. From this study, the authors suggested that bottles of SDF solutions for clinical application should be used within 60 days following their opening.

Notably, since the pKa value of NH_3_ is 9.25, the buffering effect of human saliva at ca. pH 7.0, or that of other oral fluids or environments, some of which are more acidotic (for example, pH 4.5 for primary root carious lesions), should be sufficient to release this Ag(I)-stabilising ligand from Ag(I)-coordination, and hence its liberation from SDF, will not necessarily require ligand-substitution reactions with complexing biomolecules such as amino acids and thiols with amine N-, carboxylate or phenolic O-, and/or thiol S-donors.

### 4.4 Potential tooth-demineralising properties of acidic SF products for clinical use

A further serious concern is the very low pH values of commercially-available SF products. In dental caries, lowered plaque pH values (4.5–5.5) significantly alter the saturation status of calcium and phosphate ions in the surrounding oral fluid medium (54, 55); indeed, significantly lowered pH values require higher levels of these minerals to attain saturation regarding hydroxyapatite. This is commonly known as the “critical pH value”, and represents the equilibrium extremity threshold value. For hydroxyapatite, this value is ca. 5.5, whereas for fluorohydroxyapatite, it is ca. 4.5, although it should be noted that these values have a significant “between-participant” variability (56). Hence, at pH values below this 5.5 threshold, demineralization proceeds, whereas above it, remineralization takes place. Notwithstanding, this critical pH value for hydroxyapatite is known to be significantly higher for children than it is for adults. Indeed, children are more susceptible to demineralization within a more wholly acidic oral domain, along with a diminished promotion of remineralization processes at normal oral pH values (56).

Although a degree of acidulation is required to “shield” SF in clinical product solutions against hydrolysis and photodecomposition, ironically their very low pH values are likely to give rise to deleterious tooth demineralization processes, especially since this acidulation level is markedly lower than the critical pH value of 5.5 (55). However, usually only a single drop (32 µl) of the 38% (w/v) SF (and correspondingly also SDF) product solution is clinically administered to adults (19), and for SDF, this contains only an estimated 76 µmol of this agent, with equivalent amounts of Ag(I) and F^−^ (when not considering excessive amounts of the latter present).

## 5 Conclusions

In conclusion, the experiments conducted in this study have demonstrated that high concentration commercially-available SF, but not SDF, formulation solutions for the clinical arrest of dental caries, consist of the fluoride anion rapidly exchanging with Ag(I) ion on the NMR timescale, and this phenomenon is expected to exert a major effect on the overall MoA of this product, notably a possible synergistic activity between these oppositely-charged product counterpart ions, which may include an exacerbation of the ability of Ag(I) to convey F^−^ to activity sites, e.g., hydroxyapatite. Moreover, such an exchange process may serve to propagate the biochemical and/or bioactive effects of both these species, for example the microbicidal properties of Ag(I), and the hydroxyapatite-strengthening properties of F^−^, through its ability to form more lowered pH-resistant fluorohydroxyapatite. Our results also indicate that these SF products also contain F_2_H^−^ anion through a solvation reaction of HF with F^−^, and when acting as a ligand, this species may also complex Ag(I) ions. Such agents are also likely to exert a major influence on the bioactivities and therapeutic properties of SF products. Of much concern, however, is the markedly low pH value of the SF product investigated, which ironically may engender tooth demineralisation processes, and hence this potentially adverse effect should be investigated by researchers as a matter of urgency. Hence, it certainly appears that these “caries-arresting” SF products, which are unfortunately below the critical demineralising threshold value of 5.5, could, at least in principle, serve to cause more damage and harm than any benefits offered.

Further analysis by ^19^F NMR spectroscopy confirmed that the SDF product evaluated here contained very significant molar excesses of both fluoride counter ion and its Ag(I) ammoniacal ligand donor, and this technique also revealed that excessive levels of both these agents were available for delivery to clinical application sites from known added concentrations of this commercially-available product, an observation consistent with those made by other researchers (29, 30, 52, 53). Since caries lesions treated with SF or SDF are exposed to human saliva, which continues to have access to these sites during treatment episodes (and this despite clinical precautions applied to protect against this), this investigation then focussed on the interactions of these agents with this biofluid using ^19^F NMR, FTIR-ATR, SEM and spectrophotometric techniques. Our results demonstrated that following the primary formation of off-white silver chloride precipitates, CSNPs containing high contents of Ag and Cl are generated via the IV-SCAN process, this being both time- and light exposure-dependent. FTIR-ATR analysis confirmed that these nanoparticles were encapsulated by both high- and low-molecular-mass salivary biomolecules, the former including proteins such as albumin, the latter including phospholipids and thiocyanate anion (the thiocyanate is presumably present therein as its insoluble polymeric 1:1 Ag(I) complex). Reducing agents were not required for the synthesis of these CSNPs, however, since WMSS samples contain sufficient levels of electron-donors for this purpose, e.g., L-cysteine, further thiols, SCN^−^ and/or L-tyrosine, along with proteins with reducing cysteine residues such as the single cysteine-34 residue of human albumin. These results are clearly of much clinical significance in view of the known powerful antimicrobial and cariostatic actions of Ag/AgCl- and SF-containing nanoparticles. Indeed, the deposition of solid AgCl within dentin fissures and crevices, or porous demineralized enamel, may indeed allow sufficient time for it to develop CSNPs through electron donation from suitable salivary reductants, and/or photoreduction, and accordingly this represents an alternative primary mechanism for the deposition of dark-coloured Ag(0) deposits at these sites, rather than the initial formation of insoluble Ag(I)-phosphate species from the reactions of hydroxyapatite (or salivary phosphate) with Ag(I). The time-dependent harbouring of the nanoparticulates generated in this manner may also serve to facilitate any actions they have as caries-arresting therapeutic agents directly at these disease-active locations prior to their degradation to metallic Ag.

We also conclude that in some, but not all of the WMSS samples evaluated, fluoride anion derived from added SDF may complex salivary Ca^2+^ and/or Mg^2+^ ions to form largely- and marginally-insoluble CaF_2_ and MgF_2_ products, and that thereafter, some of these metal ions may transfer to inorganic phosphates, and/or organic phosphate biomolecules, which are present in the CSNP materials isolated.

## Data Availability

The raw data supporting the conclusions of this article will be made available by the authors, without undue reservation.

## Footnotes

^1^Hunwin K, Page G, Edgar M, Botana A, Armitage R, Bhogadia M, et al. Explorations of the chemical constitution and aqueous solution status of cariesarresting silver(I)-diammine fluoride and silver(I)-fluoride products using highresolution 19F NMR analysis. Spectroscopic and SEM investigations of their interactions with human saliva: evidence for the in vivo salivary-catalysed autoconstruction of Ag/AgCl-based nanoparticles (IV-SCAN)–part II. *Front Oral Health*. (2024).
